# Supplementary material for: Design considerations for the migration from paper to screen-based media in current health education for older adults: a scoping review
Source: BMJ Open. 2024 Apr 10;14(4):e078647. doi: 10.1136/bmjopen-2023-078647 (PMC11015264; doi:10.1136/bmjopen-2023-078647)
Supplement: Author's manuscript [file bmjopen-2023-078647.draft_revisions.pdf]

BMJ Open is committed to open peer review. As part of this commitment we make the peer review history of every article we publish publicly available.

When an article is published we post the peer reviewers' comments and the authors' responses online. We also post the versions of the paper that were used during peer review. These are the versions that the peer review comments apply to.

The versions of the paper that follow are the versions that were submitted during the peer review process. They are not the versions of record or the final published versions. They should not be cited or distributed as the published version of this manuscript.

BMJ Open is an open access journal and the full, final, typeset and author-corrected version of record of the manuscript is available on our site with no access controls, subscription charges or pay-per-view fees (http://bmjopen.bmj.com).

If you have any questions on BMJ Open's open peer review process please email info.bmjopen@bmj.com

BMJ Open

Design considerations for the migration from paper to screen media in current health education for older adults: A scoping review

Journal:	*BMJ Open*
Manuscript ID	bmjopen-2023-078647
Article Type:	Original research
Date Submitted by the Author:	07-Aug-2023

Complete List of Authors:	Ferraz, Larissa; University of Surrey, Music and Media<br>Santos, Anna Julia ; Federal University of São Carlos, Department of Gerontology<br>Lorenzi, Lorena; Universidade Federal de São Carlos, Gerontology<br>Frohlich, David ; University of Surrey, Department of Music and Media<br>Barley, Elizabeth; University of Surrey, Mental Health Sciences and Nursing<br>Castro, Paula; UFSCar, Gerontology

Keywords:	Health Education, Aged, BIOTECHNOLOGY & BIOINFORMATICS, Health informatics < BIOTECHNOLOGY & BIOINFORMATICS, PUBLIC HEALTH, Primary Prevention

SCHOLARONE™
Manuscripts

BMJ

I, the Submitting Author has the right to grant and does grant on behalf of all authors of the Work (as defined in the below author licence), an exclusive licence and/or a non-exclusive licence for contributions from authors who are: i) UK Crown employees; ii) where BMJ has agreed a CC-BY licence shall apply, and/or iii) in accordance with the terms applicable for US Federal Government officers or employees acting as part of their official duties; on a worldwide, perpetual, irrevocable, royalty-free basis to BMJ Publishing Group Ltd ("BMJ") its licensees and where the relevant Journal is co-owned by BMJ to the co-owners of the Journal, to publish the Work in this journal and any other BMJ products and to exploit all rights, as set out in our licence.

The Submitting Author accepts and understands that any supply made under these terms is made by BMJ to the Submitting Author unless you are acting as an employee on behalf of your employer or a postgraduate student of an affiliated institution which is paying any applicable article publishing charge ("APC") for Open Access articles. Where the Submitting Author wishes to make the Work available on an Open Access basis (and intends to pay the relevant APC), the terms of reuse of such Open Access shall be governed by a Creative Commons licence – details of these licences and which Creative Commons licence will apply to this Work are set out in our licence referred to above.

Other than as permitted in any relevant BMJ Author's Self Archiving Policies, I confirm this Work has not been accepted for publication elsewhere, is not being considered for publication elsewhere and does not duplicate material already published. I confirm all authors consent to publication of this Work and authorise the granting of this licence.

For peer review only

TITLE PAGE

Design considerations for the migration from paper to screen media in current health education for older adults: A scoping review

Larissa Taveira Ferraz[1]

Anna Julia Tavares Santos[2]

Lorena Jorge Lorenzi[3]

David M. Frohlich[1]

Elizabeth A. Barley[4]

Paula Costa Castro[2]

1. Department of Music and Media, University of Surrey, Guildford, United Kingdom.
2. Department of Gerontology, Federal University of São Carlos, São Carlos, Brazil.
3. Programa de Pós-graduação Interunidades em Bioengenharia, University of São Paulo, São Carlos, Brazil.
4. School of Health Sciences, University of Surrey, Guildford, United Kingdom

Corresponding author:
Larissa Taveira Ferraz

Digital World Research Centre, Room 15AP01, University of Surrey, Guildford GU2 7XH, UK

Keywords: Health education, Older people, Paper-based media, Screen-based media, Review.

Word count: 6478

For peer review only

Design considerations for the migration from paper to screen media in current health education for older adults: A scoping review

ABSTRACT

Objective: To map the current use of paper-based and/or screen-based media for health education aimed at older people. **Methods**: A scoping review was carried out following the PRISMA-ScR. The search was carried out in seven databases, with studies available in the last 10 years, in English, Portuguese, Italian or Spanish, in addition to a Google Scholar search to check the grey literature. The terms used in the search strategy were older adults, health education, paper and screen-based media, preferences, intervention and other related terms. Studies were included that carried out health education interventions for older individuals using paper and/or screen-based media and that described barriers and/or facilitators of the use of these media. The selection of studies was carried out by two reviewers, in five stages. **Results:** The review included 21 studies that carried out health education interventions with different purposes, the main ones being promotion of physical activity, hypertension prevention and psychological health. All 21 interventions involved screen-based media on computers, tablets, smartphones and laptops, while only 4 involved paper-based media such as booklets, brochures, diaries, flyers and drawings. This appears to reflect a transition from paper to screen media for health education for an older population, in research if not practice. However, analysis of facilitators and barriers to use of both media revealed 10 design factors that could improve or reduce use, and complementarity in their application to each media type. For example, screen media could have multimedia Content, additional Functionality and interactivity through good Interaction Design, but have low Accessiblity and require additional Learning due to complex Interface Design. Conversely, paper media had static Content and low Functionality but high Accessibility, Availability and a low Learning overhead. Conclusions: We recommend improved screen media design, continued use of paper, and the possible combination of both media through the new augmented paper technology.

Registration number: Open Science Framework (DOI: DOI 10.17605/OSF.IO/GKEAH)

Strengths and limitations of this study

- It is the first review that mapped the differences in the use of paper-based and screen-based media for health education for older people.
- The studies were searched in seven databases, in addition to a grey literature search.

For peer review only

- The studies were verified in four languages, being Portuguese, English, Spanish and Italian.
- A limitation of the review was to have as an inclusion criterion that the studies should have a mean age equal to 60 years or older.

INTRODUCTION

The population of older adults aged 60 and overreached about 14% of the world's population in the year 2020, which means 1 in 7 people. In the year 1950, this percentage was 8% of the population.[1] Population ageing has been triggered by a series of factors, such as a decrease in the fertility rate and an increase in life expectancy.[2] The projection for 2050 is that adults over 60 will reach 22% of the world's population, that is, approximately 1 in 5 people.[1] This increase is also leading to more people living with chronic health conditions and a need to improve quality of later life through what is called healthy ageing.

The document entitled "Decade of Healthy Ageing", by the World Health Organization (WHO), proposes a plan to improve the health of the older adult population between 2021 and 2030.[3] It emphasises the importance of health conditions being managed efficiently with health promotion and disease prevention practices.[3] A key part of this strategy is the provision of effective health communication, through media that are disseminated both on paper and screen. According to WHO, health communication can be defined as "the use of communication strategies (eg, interpersonal, digital and other media) to inform and influence decisions and actions to improve health".[4]

Paper-based media are established formats for health education provided by professionals due to their widespread accessibility.[5] Such media were, and still are, widely used in health practices in the form of booklets, leaflets, posters and manuals.[6] In the literature, there are some studies that present recommendations and guidelines for their use, such as the one carried out by Hoffmann and Worrall (2004)[5], They emphasise the importance of these materials being: objective and updated; easy to understand for everyone; using table of contents, subheadings, bulleted lists, and abstracts; organised, with differentiation between uppercase and lowercase letters and use of bold only for emphasis; with the use of images or drawings only to aid understanding; and with the use of interaction resources, for example a short questionnaire.

Increasingly, health education materials are being disseminated through applications and websites on screen-based devices such as smartphones and computers.[7] Many of these

For peer review only

health education services are targeted at older adults, because they are disproportionately affected by illness and are likely to benefit most from information about healthy ageing practices. It should also be noted that, along with technological advances, there are other situations that enhance the use of digital media, such as the advent of the COVID-19 pandemic and the necessary social distancing.[8] Recommendations on the use and design of screen-based health information are also available in the literature. For example, the study by Zhao, Zhao and Song (2022)[9] explores the information seeking behaviour of older adults for health information and reveals that in addition to personal barriers, there are barriers in technology, such as unsatisfactory interactivity and usability, inadequate font sizes, dense texts, lack of visual elements and confusing layouts.

Current studies, including reviews, of the use of paper or screen formats for health information are carried out separately and seldom compared with each other. Furthermore, they address a wide range of ages in the audiences involved. In this review we aim to collect together studies of the use of both paper and screen-based media for health education targeted at older people aged 60 and over. This should allow us to compare the relative strengths and weaknesses of each media for this population and to add to the understanding of barriers and facilitators to their use. We aim to reflect on the implications of this review for the general migration of health information from paper to screen and to make concrete design recommendations for the field.

METHODS AND ANALYSIS

This review was developed according to the guidelines of the *Preferred Reporting Items of Systematic Reviews and Meta-analyses for Scoping Reviews* (PRISMA-ScR)[10] and the recommendations of the Joanna Briggs Institute Revisions Manual (JBI).[11] The protocol of the review with methodological details is published.[12] The research questions were:

1. What types of paper-based and screen-based media are used in current forms of health education and how are they used for health education of older adults?
2. What are the barriers and facilitators to using paper-based and screen-based media for health information of older adults?
3. What are the differences between the use of paper-based and screen-based media for health education?

For peer review only - http://bmjopen.bmj.com/site/about/guidelines.xhtml

Eligibility, inclusion and exclusion criteria

Scientific articles that had as their objective or central theme a health education intervention carried out with paper and/or screen-based media for health promotion or prevention of older individuals were considered eligible. Studies were included that carried out or described health education interventions for health promotion or prevention of older individuals using paper and/or screen-based media and that described barriers and/or facilitators of the use of these media. The studies included should have a sample of subjects with a mean age of 60 years or older and be published in English, Portuguese, Spanish or Italian, from 2012 until the search date. In addition, grey literature studies were included through an additional search in the Google Scholar.

Studies that were not about health promotion or prevention, such as interventions on treating or caring for pre-existing health conditions were not included. Political documents, technical reports and studies whose full texts could not be obtained, even after attempts to contact the authors, were excluded.

Search Strategy

A search strategy was developed based on preliminary searches in databases, using the terms *older adults, health education, paper-based media, screen-based media, experience, intervention*, in addition to other related terms. The search strategy was adapted using Boolean operators and Mesh terms according to each database. The search strategy used in Scopus databases was:

("older person" OR elder* OR "older adults" OR "elderly population" OR "older people" OR ageing OR aging OR "older population" OR geriatric OR "healthy ageing" OR "successful aging") AND ("paper based media" OR "screen based media" OR website* OR platform* OR virtual OR online OR multimodal OR multimedia OR "reading media" OR "digital-based reading" OR "paper based reading" OR flyer OR "media advertisement" OR "print media" OR app OR apps OR tablet* OR smartphone* OR m-health* OR e-health* OR "patient information leaflets") AND ("health education" OR "health information" OR "health communication" OR "health promotion") AND (preference* OR characteristic* OR experience* OR attribute* OR perception* OR development OR barrier* OR facilitator* OR opportunities OR problem* OR recommendations) AND (trial OR intervention)

Searches were performed in seven databases: Scopus (Elsevier), Web of Science (Clarivate), Medline, Embase (Elsevier), Cinahl (EBSCO), The ACM Guide to Computing Literature, Psyinfo (APA) databases. Studies published in Portuguese, English, Spanish and Italian, from 2012 to the date of the search in June 2022, were searched. An additional search

For peer review only - http://bmjopen.bmj.com/site/about/guidelines.xhtml

on Google Scholar was performed to verify 150 studies according to Google's relevance algorithm in the year 2022. Later, in April 2023, after guidance from the protocol reviewer that suggested the search should include the first 300 studies, based on the paper by Haddaway NR (2015),[13] the expansion of the grey literature was carried out, updating the search for April 2023, until the first 300 studies from Google Scholar were identified.

Study/Source of evidence selection

The selection of studies was carried out in five stages, starting with the search in the databases in which the identified studies were extracted into the Excel program and duplicates were removed. The next step was a pilot test in which two reviewers checked 25 random studies found in the search according to the eligibility criteria, obtaining a 92% agreement rate.

The third step was the reading of abstracts, titles and keywords of all studies by two reviewers independently following the eligibility criteria. And the fourth step was the reading of the full texts of the studies accepted in the previous step by the reviewers independently according to the inclusion criteria. In both phases, a third reviewer resolved disagreements. The last step was the additional strategy, which was the reading of the first 300 studies found in Google Scholar according to the relevance algorithm.

Data extraction

Data from the studies included in the review were extracted into a table developed and checked by the authors in three studies. The extracted data contained information about the studies such as authors' names, title, year of publication, country of origin and methods used, information about the sample such as size, gender and mean age, characteristics of the intervention such as duration and frequency, health condition verified, paper media used, screen media used, barriers to the use of paper and/or screen media and facilitators in the use of paper and/or screen media. The extraction table was described in the review protocol, however there was a change regarding the topic "is the health education aimed at the caregiver?" which was withdrawn, as the target population of the review study is adults 60+ regardless of occupation.[12]

Data analysis and presentation

The data extracted from the included studies were presented in a narrative form and through tables and the PRISMA flowchart. Quantitative data were analysed using relative and absolute frequency, mean and standard deviation. Qualitative data about the barriers and

For peer review only - http://bmjopen.bmj.com/site/about/guidelines.xhtml

facilitators in the use of paper and/or screen-based media were analysed using Bardin's content analysis.[14] Barriers and facilitators were grouped into categories according to their similarity.

RESULTS

The databases searched resulted in 1,392 studies, as shown in the flow diagram in figure 1. Duplicate studies were removed which resulted in 1,080 articles found. All the studies found were screened by the two reviewers, and according to the eligibility criteria, a total of 134 articles were sent to the reading step of the full texts. In the selection of studies by the inclusion criteria the total number of included studies was 15 and 119 were excluded as described in the flowchart (Figure 1). In the additional strategy, which was the reading of the 300 studies found in Google Scholar in order of relevance, it was identified that 21 were duplicates that had already been found in the search of the databases, thus 321 studies were identified in order to have 300 studies without duplicates to be screened for eligibility. As a result, 95 studies were selected for full reading and, subsequently, 6 were included based on the same inclusion criteria applied to full texts. Thus, both searches performed had a total of 21 studies included.

In the search in the databases and in the additional strategy, a study was excluded for these reasons: it does not present the mean age or the mean age is less than 60 years; it does not present barriers or facilitators perceived by users in relation to the media used; it does not have a health education or the health education is not for promotion or prevention.

Figure 1: PRISMA flow diagram of the studies included in the review that presents screen-based and/or paper-based media for health education.

(FIGURE 1)

Description: Reason 1: it is a protocol, literature review or it has only an abstract available; Reason 2: it does not present the mean age or the mean age is less than 60 years; Reason 3: it does not have participants; Reason 4: it does not have a health education or the health education is not for promotion or prevention; Reason 5: health education does not use paper or screen-based media; Reason 6: it does not describe the health education; Reason 7: it does not present barriers or facilitators perceived by users in relation to the media used.

Source: developed by the authors.

For peer review only

Characterization of the studies

Descriptive characteristics of the included studies (n = 21), such as year of publication, country where it was carried out, year of data collection, methodology, intervention, sample size and average age of participants are described in Table 1.

Table 1 – Descriptive characteristics of the included studies

**Variables**	**n**	**%**
Year of publication		
2012	1	4.76
2013	1	4.76
2014	2	9.52
2015	2	9.52
2016	2	9.52
2017	2	9.52
2018	5	23.81
2019	2	9.52
2021	3	14.29
2022	1	4.76
Location - Country		
United States	5	23.81
Australia	3	14.29
Netherlands	3	14.29
New Zealand	2	9.52
South Korea	2	9.52
Belgium	1	4.76
Malaysia	1	4.76
Germany	1	4.76
Taiwan	1	4.76
International (several countries)	2	9.52
Year of data collection		
2007-2009	1	4.76
2010-2011	1	4.76
2012	2	9.52
2014-2015	1	4.76
2015	1	4.76
2016	1	4.76
2017	1	4.76
2017-2018	1	4.76
2020	1	4.76
2020-2021	1	4.76
NR	10	47.62
Methodology		
Longitudinal	17	80.95

For peer review only

Cross-sectional	4	19.05
Intervention		
In-person	11	52.38
Online	2	9.52
In-person and online	8	38.10
Sample size		
05-30	6	28.57
31-60	7	33.33
84	1	4.76
121-150	4	19.05
204	1	4.76
218	1	4.76
703	1	4.76
Average age of participants		
60-64 years	5	23.81
65-69 years	7	33.33
70-74 years	4	19.05
75-79 years	3	14.29
Unknown	2	9.52

NR: Not reported
Source: developed by the authors.

Regarding the year of publication described in table 1, approximately 61,9% of the articles were published in 2017, 2018, 2019, 2021 or 2022. Respectively, the United States, Australia, Netherlands, New Zealand and South Korea were the countries that most originated studies in this review. The year of data collection in the studies showed great variability, with the oldest year being 2007 and the most recent year being 2021. The majority of studies were carried out longitudinally (80.95%).

The interventions were mostly face-to-face (52.38%) and had a minimum duration of 30 minutes and a maximum duration of 6 months. The studies' sample ranged from 5 to 703 participants, however, mostly had 31 to 60 participants (33.33%) or 5 to 30 participants (28.57%) and most of the studies had an average age between 65 to 69 years old (33.33%) or 60 to 64 years old (23.81%). Two studies (9,52%) did not report mean age, but did not have adults (aged 18 to 59) in the sample. The mean total age of participants across all included studies was 68.51 (±5.06) years. Most studies (90.48%) were performed with individuals of both genders and the other studies did not report data on gender. In addition, 47,62% of the studies did not have adults (aged 18 to 59) in the sample, 47.62% of the studies had adults, and one study did not report this information, but the mean age was over 60 years.

Health education themes and types of media found in the studies.

For peer review only

In response to the first research question of this review about the *formats* of health information used and for what purposes, Table 2 presents all the studies included in this scoping review, with their authors, year of publication, central theme of the health education and what type of digital/ paper media format was used.

The central theme of health education had great variability among the included studies, however it was identified that the themes that most appeared were physical activity promotion (14.29%), psychological health (14.29%) and hypertension prevention and care (9.52%).

Among the screen-based media formats used for health education in the studies, the following media were identified: website (61.90%), app (23.81%), email (9.52%) and animations on Microsoft PowerPoint (4.76%). These media were used on the devices: computers (52.38%), tablets (28.57%), smartphones (19.05%) and laptops (4.76%). Some studies used more than one media or device. Regarding the paper-based media formats used for health education in the studies, it was identified that they were booklets (A4 Paper), brochures, handouts, diaries, flyers, drawings and print-delivered information (without specification).

A major finding of the review was the imbalance of studies on paper and screen interventions. All 21 articles included in this study featured screen-based media, and among these, only 4 also reported the contrasting use of paper-based media. No studies were found that reported on the use of paper-based interventions alone.

Table 2: Studies included in the review, health education provided and types of paper-based and/or screen-based media used.

Article title	Authors, Year	Central theme of the health education	Screen-based Media Format	Paper-based Media format
A Digital Human for Delivering a Remote Loneliness and Stress Intervention to At-Risk Younger and Older Adults During the COVID-19 Pandemic: Randomized Pilot Trial [15]	Loveys et al., 2021	Mental health and well-being	Computer, tablet or smartphone (website)	NA (Not applicable)
A Multi-faceted Approach to Promote Comprehension of Online Health Information Among Older Adults [16]	Chin et al, 2018	Self-care regarding hypertension	Computer	NA
Acceptability of a guided self-help Internet intervention for family caregivers: Mastery over dementia [17]	Pot; Blom; Willemse, 2015	Psychological distress of family caregivers of people with dementia	(Website)	NA

For peer review only

Assessing the Impact of a Game-Centered Mobile App on Community-Dwelling Older Adults' Health Activation [18]	Crandall; Shake; Ziegler, 2019	Risk of falls and osteoarthritis	Tablet (App)	NA
Co-design of an evidence-based health education diabetes foot app to prevent serious foot complications: a feasibility study [19]	Ogrin et al, 2018	Foot health for people with diabetes	Smartphone (app)	NA
Development and assessment of a web-based intervention for educating older people on strategies promoting healthy cognition [20]	Vanoh et al, 2018	Precautionary strategies for mild cognitive impairment	Computer (Website)	NA
Development and Evaluation of a Computerized Multimedia Approach to Educate Older Adults about Safe Medication [21]	Im & Park, 2014	Safe medication	Computer (Website)	NA
Development and Validation of an Interactive Internet Platform for Older People: The Healthy Ageing Through Internet Counselling in the Elderly Study [22]	Jongstra et al, 2017	Improving the cardiovascular risk profile of old people with high cardiovascular risk	Computer (Website)	NA
Development of a communication intervention for older adults with limited health literacy: Photo stories to support doctor–patient communication [23]	Koops et al., 2016	Doctor-patient communication and communicative health literacy	(Website)	Booklet (A4 Paper)
Differences in the use and appreciation of a web-based or printed computer-tailored physical activity intervention for people aged over 50 years [24]	Peels et al, 2013	Physical activity promotion	Computer (Website; email)	Print-delivered information, brochures and handouts
Efficacy of a web-based, center-based or combined physical activity intervention among older adults [25]	Mouton & Cloes, 2015	Physical activity	(Website; email)	NA
Evaluation of the usability and acceptability of the InnoWell platform as rated by older adults: Survey study [26]	Lamonica et al 2021	Biopsychosocial domains	Tablet, computer, laptop, smartphone (Website)	NA
Improving older adults'e-health literacy through computer training using NIH online resources [27]	Xie; Bo, 2012	Improve the ability of seniors to search, find and understand health information online	Computer (website)	NA

For peer review only

Mobile App Use for Insomnia Self-Management in Urban Community-Dwelling Older Korean Adults: Retrospective Intervention Study [28]	Chung et al, 2018	Sleep education	Smartphone (app)	NA
Self-efficacy, self-regulation, and physical activity behavior in type 2 diabetes [29]	Olson, 2014	Metabolic Health Education	(Website)	NA
The Application of Virtual Reality in Patient Education[30]	Pandrangi et al, 2019	Health education - health status	(Website)	Flyers, drawings and brochures
The GezelschApp A Dutch Mobile Application to Reduce Social Isolation and Loneliness [31]	Jansen-Kosterink, Varenbrink & Naafs, 2018	Social isolation and loneliness among old people	Computer and tablet (App)	NA
The Impact of 3-D Models versus Animations on Perceptions of Osteoporosis and Treatment Motivation: A Randomised Trial[32]	Jones et al, 2017	Information about osteoporosis	Tablet (animations on Microsoft PowerPoint)	NA
The influence of robot-assisted learning system on health literacy and learning perception [33]	Wei et al, 2021	Hypertension prevention for older adults	Tablet and computer	NA
Web-based oral health promotion program for older adults: Development and preliminary evaluation [34]	Marino et al, 2016	Oral health promotion	Computer (Website)	NA
Web-based versus print-based physical activity intervention for community-dwelling older adults: crossover randomized trial [35]	Pischke et al., 2022	Promoting a physically active (PA) lifestyle	(Website and app)	Brochures and diary

NA: Not applicable.
Source: developed by the authors.

For peer review only

Categorization of barriers and facilitators

In response to the second research question about the barriers and facilitators to use, it was identified that the vast majority were about screen-based media. Thus, they were grouped according to their similarity into 10 categories, described below:

- Accessibility - the diversity of users able to access the media system.
- Availability - the availability to use the media system.
- Content design - the design of the media content itself.
- Functionality - what the media system does.

- Interaction design - design of the sequential organisation of the interaction with the media system.
- Interface design - design of the input and output interfaces.
- Learning - the ease of learning to use the media system.
- Privacy - privacy of personal data in the media system.
- Social interaction - the support of interaction with other people around the media system.
- Usability - the ease of using the media system.

The topics defined for the categorisation of barriers and facilitators seem to be properties of the design of media systems in general that can have both negative (barriers) and positive (facilitators) effects. They are different sides to the same coin because if done well they improve health education, but if done badly they reduce it. The categories found in barriers and facilitators related to screen-based and paper-based media are described in Table 3. The table describes the number of studies that identified each category, with some studies finding more than one type of category as barriers and/or facilitators. In the following sub-sections we step through the cited barriers and facilitators for each medium in turn.

Table 3 - Barriers and facilitators found in the included studies.

Topics	Screen-Based Media		Paper-Based Media	
	**Barriers**	**Facilitators**	**Barriers**	**Facilitators**
**Accessibility**	5	3	--	--
**Availability**	1	3	--	2
**Content design**	4	8	--	1
**Functionality**	3	6	2	--
**Interaction design**	2	4	--	--
**Interface design**	2	4	--	--
**Learning**	6	4	--	--
**Privacy**	1	--	--	--
**Social interaction**	1	2	--	--
**Usability**	3	6	--	--

Source: developed by the authors.

For peer review only

Barriers of screen-based media

The main categories that appeared as barriers to the use of screen-based media were Learning (e.g. lack of computer training, lack of instructional videos, clear guidelines, technology training to be completed in-person), Accessibility (e.g. would prefer a larger screen using a tablet or laptop, hard copies of the information, a sad thing is you can't touch her or talking to a real person), Content design (e.g. strange contents, technical terms, information wasn't as up to date, participants wanted varied levels of information), Functionality (e.g. lack of reward, some technical improvements are needed like the function to delete messages, defining a goal was difficult for the participants, an icon to facilitate printing would be useful) and Usability (e.g. the assignments were not direct and required big effort, passwords provided to login for the first time were too difficult). However, the following barriers were also identified: Availability (e.g. computer availability and computer reserves), Interaction design (e.g. aspects participants felt could be improved were the human likeness of it interaction like behaviours and voice and aspects of the conversation design), Interface design (e.g. videos were not easy to see because they had small size, small font size, lack of attractive photos and project colours), Privacy (e.g. participants did not post on the discussion board due to privacy concerns), Social interaction (e.g. limited app interaction with participants).

Facilitators of screen-based media

The main categories that appeared as facilitators for the use of screen-based media were Content design (e.g. information with revised content by researchers and experts helped them to understand the content, easy-to-understand text structures, bulleted lists, and phrases, animation based on a life story of an older adult character contributed to enhancing the self-efficacy of the participants), Usability (e.g. operating the program just by clicking the mouse was easy, it was simple and easy to navigate on a mobile phone, easy to use interface, most participants found these sites easy or very easy to use), Functionality (e.g. participants really liked the function to be able to make new friends and send and receive messages, participants mentioned videos as the most useful components, feedback was helpful), Accessibility (e.g. the app was appropriate for people with a low level of education, touch screen facilitates the use by old adults) and Interaction design (e.g. participants appreciated the interactive features of the platform like the interactive videos, interactive questions help with problem-solving, participants liked the interpersonal skills). However, the following facilitators were also identified: Availability (e.g. information on the smartphone can be seen at any time quickly, permanent access to the website), Interface design (e.g., the animations on the tablet allowed

For peer review only

more of the information to be visualised, participants liked the appearance, cross-referenced a number of the images and statements to various sections), Learning (e.g. participants reported that they easily learned how to use the application, older adults required technology training to be completed in-person, they also enjoyed and felt comfortable with self-learning), Social interaction (e.g. many participants expressed how much they enjoyed the social aspect of the app, participants considered the app a pleasant and safe way to find new friends).

Barriers of the use of paper-based media

Regarding the barriers related to paper-based media, the study by Pischke et al (2022)[35] found Functionality to be a barrier as the printed version seemed to be impractical regarding physical activity. Pandrangi et al (2019)[30] also identified Functionality as a barrier since participants perceived paper as a traditional method and considered virtual reality more useful.

Facilitators of the use of paper-based media

In the study by Koops et al. (2016)[23] it was identified that the paper was a facilitator in relation to Availability, as the participants reported that they could take it anywhere and Content design, as they liked that the stories were similar to real life and that it was easier for them to understand. Peels et al. (2013)[24] found availability to be a facilitator since the participants had more intentions to keep the information and to use the concepts learned in the future.

Differences between the use of paper-based and screen-based media for health education

Regarding the third research question about the differences between the use of paper-based and screen-based media, this was difficult to answer because of the small number of direct comparisons made. However, three particular barrier/facilitator categories appeared to relate to the contrasting properties of paper and screen, as expressed in the four studies using paper and screen materials. These were Availability, Functionality and Learning.

Availability appeared in two studies as facilitators concerning the use of paper media due to the possibility of having the paper in hand and being able to use it at any time. For the use of screen-based media, there is a need for more effort, for example, when having to turn on

For peer review only

a computer, log in to a website, download an application, etc., in order to be able to start using this media.[23, 24]

Considering the use of paper-based media, two other studies identified Functionality as a barrier to their use, due to the fact that this is limited compared to screen media, in which there is the possibility of having different functions, such as having feedback, explanatory videos, setting goals and being able to make new friends.[30, 35]

In addition, the Learning category is related to the difference in the use of paper and screen due to the older audience having individuals who have difficulty using screen media, as they have no affinity with it and need training to use them.[35, 25] Thus, for individuals with difficulty in relation to the use of screen media, the use of paper is preferable, as seen in the study by Pischke et al. (2022).[35]

DISCUSSION

The review identified 21 studies that carried out health education interventions to promote ways of staying healthy longer to an ageing population subject to more chronic health conditions, the main subjects in the interventions were related to promotion of physical activity, hypertension prevention and psychological health. This was reflected in the health education subjects of the papers . These were directed to issues such as metabolic health, sleep, healthy eating, physical activity and cognition, and focused on the development and maintenance of autonomy and independence, enabling healthy ageing, according to the WHO definition.[3]

The interventions were done through the use of screen-based media such as websites, apps and emails, on desktop computers, tablets, smartphones and laptops, and paper-based media such as booklets, brochures, diaries, flyers and drawings. We found that the barriers and facilitators to the use of these media were essentially design properties that could improve media use if done well or reduce use if done badly. This led us to propose a taxonomy of 10 design features and considerations which apply to both media, somewhat unequally. The main design considerations for the use of screen-based media were related to Content design, Functionality, Usability, Accessibility and Learning. The main design considerations for the use of paper-based media, were Availability and Content design, but such media were often considered traditional because they did not have extra functionality.

We can see from the diversity of paper and screen materials in the studies that these terms each refer to quite different formats. In fact, they gloss these differences and do not really do justice to the different interactions people could have with each format. This may be one

reason for the ambiguity of findings in such comparisons in the past, and suggests greater attention to *particular* paper and screen formats in future research. For example, screen information was presented on four physical devices in the above studies, including desktop and laptop computer, tablet and smartphone. Sometimes this information was on a website or presented in a more interactive app, leading to a matrix of 8 possibilities across devices with different user experiences. Likewise, paper information was presented on single page leaflets, fliers and handouts, as well as multi-page booklets or diaries. Each of these has different affordances for reading, finding, handling and capturing information, and are not equivalent user experiences. Further work is required to understand the subtleties of design, interaction and experience will all these ‘paper’ and ‘screen’ formats, since the current papers do not go into sufficient detail.

Regarding the types of digital media used for health education, the World Health Organization has a 'Global Strategy on Digital Health 2020-2025'[36] with the purpose of strengthening health systems through digital health technology and this will engage patients to achieve the vision of health and well-being for all. This strategy signals that practices for public awareness of digital health must be addressed, this includes health literacy and patient engagement.[36] Our findings clearly reveal a noticeable trend towards digitization and a preference for screen-based media in the literature. When analysing the review data from the last decade, we observed a significant increase in the number of studies and publications focusing on screen-based media across all years, as depicted both in Tables 1 and 2. Our study found barriers and facilitators that, if properly addressed, will make new digital health strategies more user friendly for older people, which will improve the engagement of this group in health education interventions and consequently improve health literacy. Key amongst these were the design of *functionality* and *content*, which were shown to have a great impact, both positive and negative, on interventions.

Regarding screen media use, learning to use the media was one of the most prevalent barriers. According to De Paiva and Alves (2018)[37], the current population of older users accompanied the emergence of the internet, the printer and the computer. The younger generations, such as those born in 1980-2010, grew up adapting to new technologies, as they already existed when they were born.[37] Nowadays, with a technological generation, games with different levels of proficiency and the emergence of artificial intelligence, the older population finds it difficult to adapt. Thus, older people have greater difficulty with screen-based media and require more training.

For peer review only

Regarding the use of paper media for health education, according to the findings of this review, it is clear that these media are still very much alive today, as revealed in the study by Koops et al. (2016).[23] For example, a booklet with photographic stories that aided in the communication between doctor and patient was considered to have good availability, as it could be given out and used anywhere. This is likely to reflect a pervasive practice of displaying posters and giving out leaflets in GP surgeries and hospitals across the globe, regardless of their lack of representation in this research review. Indeed, this is mentioned in the wider literature. For example Maskell, McDonald and Paudyal (2018)[38] studied the use of printed materials used in the waiting rooms of general clinics in England and found that they were noticed by more of three-quarters of patients, and read by a large proportion of them. Hence, paper-based media are already widely accepted and available as part of modern health education practices.

Concerns about data privacy were cited in the study by Olson (2014)[29] in this review. Despite being mentioned by only one of the studies, privacy is an important issue for the proliferation of screen-based information compared with paper. This is because digital engagement carries the danger and opportunity of data capture and user profiling. The Global Strategy on Digital Health 2020-2025[36] emphasises that health data is sensitive and personal, it requires a high standard of security and protection. Lack of trust and confidence about personal health data can discourage people from fully participating in health education interventions. WHO mentions the need for a strong legal and regulatory foundation to protect privacy, integrity, confidentiality and deal with data cyber security. It is also important to maintain transparency and communicate effectively about data security strategies to users.[36]

Regarding the international reach of these studies we note that they come largely from high income countries as shown in Table 1. There is a lack of research and representation from low and middle income countries, especially those with vulnerable populations, and large countries with vast and remote regions facing challenges in accessing healthcare services, as shown in Table 1. For populations with limited education and financial vulnerability, paper-based materials appear more accessible due to their traditional usability and cost-effectiveness. However, for individuals who are actually illiterate, screen readers and audio content, for instance, offer a level of accessibility that paper cannot match. Moreover, advancements in satellite connection technologies are now integrating previously isolated areas into the global network, potentially granting access to health information for a substantial number of people who currently lack access to up-to-date pamphlets and leaflets. This integration and interactivity brought about by screen-based media could also enable these individuals to connect with health teams and professionals in more densely populated areas. This, in turn,

For peer review only

could lead to more personalised and humanised health education guidelines and interventions through technology. Overall, these developments hold promise in bridging the healthcare information gap and fostering improved health outcomes for underserved communities.

Given the advantages and disadvantages of paper and screen-based information emerging from this review, it may be valuable to combine them in future health campaigns, perhaps at a new level of granularity. This can be done through *augmented paper* technology which supports the reading of printed hotlinks on a nearby smartphone.[39] This results in a paper-and-screen reading experience in which users move back and forth between media which have been carefully designed to complement each other. For example, a printed travel guide can play interviews with local characters, video walks through the landscape, and trigger phone calls to organisations or show updated timetable information.[40] The user in this scenario does not have to know how to use the web to access the digital information. They simply take a picture of the printed page, speak its page number, turn the page or move a bookmark to access the links.[41] A similar approach could be taken to the design of augmented patient information leaflets, posters and cards, which could play multimedia illustrations, spoken translations or instructions, and call local clinics or medical services for more advice.[42]

This review is limited to studies reporting the use of participants with a mean age equal to 60 years or older. Thus, studies that had not reported the mean age or age range of participants were excluded, but it may be that some would have been included if they had reported this information. The strengths of this review are: this is the first study that identifies and compares digital and paper media for health education aimed at older people, as well as design considerations related to their use; studies were searched for in seven databases and in the grey literature; studies were searched for in four languages, namely Portuguese, English, Spanish and Italian; systematic methods were used for the selection of studies e.g. all titles, abstracts and full texts were screened by two reviewers independently.

CONCLUSION

A transition or migration appears to be taking place in the provision of health information from paper to screen. In this review we have examined recent literature on the design and use of such information for older people across these formats and found an over-representation of research on screen interventions. Although this suggests that the future lies in greater digital engagement, a careful reading of 21 papers shows many barriers to the use of screen-based information and corresponding facilitators to the use of paper information for health, especially for an older population and broader international context. We therefore

For peer review only

recommend greater attention to the barriers of screen-based information across different devices and services, continued use of paper, and the possible combination of both media through new augmented paper technology.

ETHICS AND DISSEMINATION

Ethical approval is not applicable to this scoping review, it only gathered information from published literature. The results will be disseminated through presentations at significant scientific events and published in journals in the area.

Author Contributions: All authors have made substantial contributions to the development of the manuscript. LTF and AJTS conceptualised the research question and prepared the first draft of the manuscript. LTF, AJTS, LJL, PCC, DMF, EAB contributed to the refining of the study design and guided the review development. Also, LTF, AJTS, and LJL analysed the results and PCC, DMF, EAB contributed to the discussion. Also LTF, AJTS, LJL, PCC, DMF, EAB contributed to the writing, and revising this review. DMF assisted in the revision of the English.

Competing interests: The authors declare no conflict of interest.

Funding: LTF received funding from the University of Surrey Doctoral College (Breaking Barriers Studentship Award), AJTS received funding from São Paulo Research Foundation (FAPESP), N° 2022/08918-6 to the development of this review and LJL received funding from Coordination for the Improvement of Higher Education Personnel (CAPES) - Financial Code 001.

Data sharing statement: Data from this review are available in this manuscript and in the previously published protocol. As it is a scoping review, the sharing of other data is not applicable.

Patient and Public Involvement statement: Not required.

Acknowledgements: We would like to acknowledge University of Surrey Doctoral College, São Paulo Research Foundation (FAPESP), Coordination for the Improvement of Higher Education Personnel (CAPES) for funding.

For peer review only

REFERENCES:

1. United Nations, Department of Economic and Social Affairs, Population Division (2022). World Population Prospects: The 2022 Revision. 2022. Available from: https://population.un.org/dataportal/.
2. United Nations Department of Economic and Social Affairs, Population Division (2022). World Population Prospects 2022: Summary of Results. 2022b. UN DESA/POP/2022/TR/NO. 3.
3. World Health Organization. Decade of healthy ageing: baseline report. Geneva, 2020.
4. World Health Organization. Health promotion glossary of terms 2021. 2021
5. Hoffmann, T., & Worrall, L. (2004). Designing effective written health education materials: considerations for health professionals. Disability and rehabilitation, 26(19), 1166-1173.
6. Sá, G. G. D. M., Silva, F. L., Santos, A. M. R. D., Noleto, J. D. S., Gouveia, M. T. D. O., & Nogueira, L. T. (2019). Technologies that promote health education for the community elderly: integrative review. Revista latino-americana de enfermagem, 27.
7. Kampmeijer, R., Pavlova, M., Tambor, M., Golinowska, S., & Groot, W. (2016). The use of e-health and m-health tools in health promotion and primary prevention among older adults: a systematic literature review. BMC Health Services Research, 16(5), 467-479.
8. Celuppi IC, Lima GS, Rossi E, et al. An analysis of the development of digital health technologies to fight COVID-19 in Brazil and the world. Cad Saude Publica 2021;37(3):e00243220. Doi: 10.1590/0102-311X00243220.
9. Zhao YC, Zhao M, Song S. Online Health Information Seeking Behaviors Among Older Adults: Systematic Scoping Review. Journal of medical Internet research 2022; 24 (2): e34790. DOI: 10.2196/34790
10. Tricco AC, Lillie E, Zarin W, et al. PRISMA Extension for Scoping Reviews (PRISMA-ScR): Checklist and Explanation. Ann Intern Med. 2018 ;169(7):467-73. Doi: 10.7326/M18-0850.
11. Peters MDJ, Godfrey C, McInerney P, et al. Chapter 11: Scoping Reviews (2020 version). In: Aromataris E, Munn Z (Editors). Joanna Briggs Institute Reviewer's Manual; 2020. Available from https://reviewersmanual.joannabriggs.org/

12. Ferraz LT, Santos AJT, Lorenzi LJ, et al. Paper and screen media in current health education practices aimed at older adults: a scoping review protocol. BMJ Open 2023;13:e068762. doi:10.1136/ bmjopen-2022-068762
13. Haddaway NR, Collins AM, Coughlin D, Kirk S (2015) The Role of Google Scholar in Evidence Reviews and Its Applicability to Grey Literature Searching. PLoS ONE 10(9): e0138237. doi:10.1371/journal.pone.0138237
14. Bardin L. Content analysis. Lisboa: Edições, 2009.
15. Loveys, K., Sagar, M., Pickering, I., & Broadbent, E. (2021). A digital human for delivering a remote loneliness and stress intervention to at-risk younger and older adults during the COVID-19 pandemic: Randomized pilot trial. JMIR mental health, 8(11), e31586.
16. Chin, J., Moeller, D. D., Johnson, J., Duwe, E. A., Graumlich, J. F., Murray, M. D., & Morrow, D. G. (2018). A multi-faceted approach to promote comprehension of online health information among older adults. The Gerontologist, 58(4), 686-695.
17. Pot, A. M., Blom, M. M., & Willemse, B. M. (2015). Acceptability of a guided self-help Internet intervention for family caregivers: mastery over dementia. International psychogeriatrics, 27(8), 1343-1354.
18. Crandall, K. J., Shake, M., & Ziegler, U. (2019). Assessing the Impact of a Game-Centered Mobile App on Community-Dwelling Older Adults′ Health Activation. OBM integrative and complimentary medicine, 4(3).
19. Ogrin, R., Viswanathan, R., Aylen, T., Wallace, F., Scott, J., & Kumar, D. (2018). Co-design of an evidence-based health education diabetes foot app to prevent serious footcomplications: a feasibility study. Practical diabetes, 35(6), 203-209d.
20. Vanoh, D., Ishak, I. H., Shahar, S., Manaf, Z. A., Ali, N. M., & Noah, S. A. M. (2018). Development and assessment of a web-based intervention for educating older people on strategies promoting healthy cognition. Clinical interventions in aging, 1787-1798.
21. Im, C., & Park, M. (2014). Development and evaluation of a computerized multimedia approach to educate older adults about safe medication. Asian Nursing Research, 8(3), 193- 200.
22. Jongstra, S., Beishuizen, C., Andrieu, S., Barbera, M., van Dorp, M., van de Groep, B., ... & Richard, E. (2017). Development and validation of an interactive internet platform for older people: the healthy ageing through internet counselling in the elderly study. Telemedicine and e-Health, 23(2), 96-104.
23. Koops van't Jagt, R., de Winter, A. F., Reijneveld, S. A., Hoeks, J. C., & Jansen, C. J. (2016). Development of a communication intervention for older adults with limited

For peer review only

health literacy: Photo stories to support doctor–patient communication. Journal of Health Communication, 21(sup2), 69-82.

24. Peels, D. A., De Vries, H., Bolman, C., Golsteijn, R. H. J., Van Stralen, M. M., Mudde, A. N., & Lechner, L. (2013). Differences in the use and appreciation of a web-based or printed computer-tailored physical activity intervention for people aged over 50 years. Health Education Research, 28(4), 715-731.
25. Mouton, A., & Cloes, M. (2015). Efficacy of a web-based, center-based or combined physical activity intervention among older adults. Health Education Research, 30(3), 422- 435
26. LaMonica, H. M., Roberts, A. E., Davenport, T. A., & Hickie, I. B. (2021). Evaluation of the usability and acceptability of the InnoWell platform as rated by older adults: survey study. JMIR aging, 4(2), e25928.
27. Xie, B. (2012). Improving older adults'e-health literacy through computer training using NIH online resources. Library & information science research, 34(1), 63-71.
28. Chung, K., Kim, S., Lee, E., & Park, J. Y. (2020). Mobile app use for insomnia selfmanagement in urban community-dwelling older Korean adults: retrospective intervention study. JMIR mHealth and uHealth, 8(8), e17755.
29. Olson, E. A. (2014). Self-efficacy, self-regulation, and physical activity behavior in type 2 diabetes. University of Illinois at Urbana-Champaign.
30. Pandrangi, V. C., Gaston, B., Appelbaum, N. P., Albuquerque Jr, F. C., Levy, M. M., & Larson, R. A. (2019). The application of virtual reality in patient education. Annals of vascular surgery, 59, 184-189.
31. Jansen-Kosterink, S., Varenbrink, P., & Naafs, A. (2018). The GezelschApp a Dutch mobile application to reduce social isolation and loneliness. In 4th International Conference on Information and Communication Technologies for Ageing Well and e-Health, ICT4AWE 2018 (pp. 142-147). SCITEPRESS.
32. Jones, A. S., Fernandez, J., Grey, A., & Petrie, K. J. (2017). The impact of 3-D models versus animations on perceptions of osteoporosis and treatment motivation: a randomised trial. Annals of Behavioral Medicine, 51(6), 899-911.
33. Wei, C. W., Kao, H. Y., Wu, W. H., Chen, C. Y., & Fu, H. P. (2021). The influence of robotassisted learning system on health literacy and learning perception. International Journal of Environmental Research and Public Health, 18(21), 11053.
34. Marino, R. J., Marwaha, P., & Barrow, S. Y. (2016). Web-based oral health promotion program for older adults: Development and preliminary evaluation. International journal of medical informatics, 91, e9-e15.
35. Pischke, C. R., Voelcker-Rehage, C., Ratz, T., Peters, M., Buck, C., Meyer, J., ... & Lippke, S. (2022). Web-based versus print-based physical activity intervention for community-dwelling older adults: crossover randomized trial. JMIR mHealth and uHealth, 10(3), e32212.
36. World Health Organization. (2021a). Global strategy on digital health 2020-2025.
37. De Paiva, D. C.; Alves, H. V.. Evolução Tecnológica e as Diferentes Gerações. 2018.[1]

For peer review only

38. Maskell, K., McDonald, P., & Paudyal, P. (2018). Effectiveness of health education materials in general practice waiting rooms: a cross-sectional study. *British Journal of General Practice*, *68*(677), e869-e876.
39. Frohlich, D. M. (2017, October). From Audio Paper to Next Generation Paper. In *Proceedings of the 23rd Brazillian Symposium on Multimedia and the Web* (pp. 9-10).
40. Frohlich D, Corrigan-Kavanagh E, Bober M, et al. The Cornwall a-book: An augmented travel guide using next generation paper. The Journal of Electronic Publishing 2019;22(1). DOI: https://doi.org/10.3998/3336451.0022.101.
41. Bairaktaris, G., Frohlich, D., & Sporea, R. (2021, May). Printed Light Tags and the Magic Bookmark: Using light to augment paper objects. In *Extended Abstracts of the 2021 CHI Conference on Human Factors in Computing Systems* (pp. 1-5).
42. Frohlich, D. M., Ferraz, L., & Castro, P. (2022). Printed digital storytelling for older audiences [2]

For peer review only

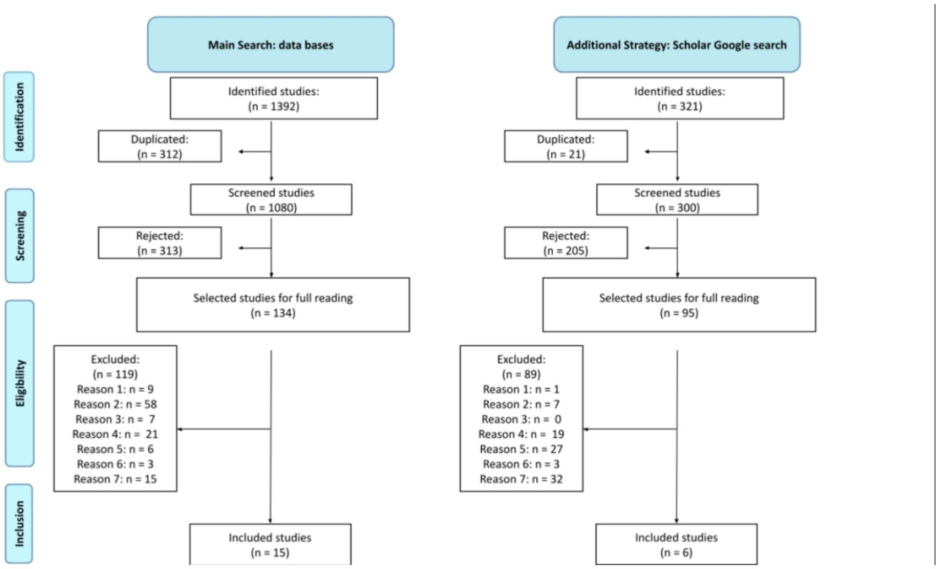

Description: Reason 1: it is a protocol, literature review or it has only an abstract available; Reason 2: it does not present the mean age or the mean age is less than 60 years; Reason 3: it does not have participants; Reason 4: it does not have a health education or the health education is not for promotion or prevention; Reason 5: health education does not use paper or screen-based media; Reason 6: it does not describe the health education; Reason 7: it does not present barriers or facilitators perceived by users in relation to the media used.
Source: developed by the authors.

234x131mm (144 x 144 DPI)

BMJ Open

Design considerations for the migration from paper to screen media in current health education for older adults: A scoping review

Journal:	*BMJ Open*
Manuscript ID	bmjopen-2023-078647.R1
Article Type:	Original research
Date Submitted by the Author:	23-Jan-2024

Complete List of Authors:	Ferraz, Larissa; University of Surrey, Music and Media<br>Santos, Anna Julia ; Federal University of São Carlos, Department of Gerontology<br>Lorenzi, Lorena; Universidade Federal de São Carlos, Gerontology<br>Frohlich, David ; University of Surrey, Department of Music and Media<br>Barley, Elizabeth; University of Surrey, Mental Health Sciences and Nursing<br>Castro, Paula; UFSCar, Gerontology

**Primary Subject Heading**:	Public health
Secondary Subject Heading:	Health policy, Health services research
Keywords:	Health Education, Aged, BIOTECHNOLOGY & BIOINFORMATICS, Health informatics < BIOTECHNOLOGY & BIOINFORMATICS, PUBLIC HEALTH, Primary Prevention

SCHOLARONE™
Manuscripts

I, the Submitting Author has the right to grant and does grant on behalf of all authors of the Work (as defined in the below author licence), an exclusive licence and/or a non-exclusive licence for contributions from authors who are: i) UK Crown employees; ii) where BMJ has agreed a CC-BY licence shall apply, and/or iii) in accordance with the terms applicable for US Federal Government officers or employees acting as part of their official duties; on a worldwide, perpetual, irrevocable, royalty-free basis to BMJ Publishing Group Ltd ("BMJ") its licensees and where the relevant Journal is co-owned by BMJ to the co-owners of the Journal, to publish the Work in this journal and any other BMJ products and to exploit all rights, as set out in our licence.

The Submitting Author accepts and understands that any supply made under these terms is made by BMJ to the Submitting Author unless you are acting as an employee on behalf of your employer or a postgraduate student of an affiliated institution which is paying any applicable article publishing charge ("APC") for Open Access articles. Where the Submitting Author wishes to make the Work available on an Open Access basis (and intends to pay the relevant APC), the terms of reuse of such Open Access shall be governed by a Creative Commons licence – details of these licences and which Creative Commons licence will apply to this Work are set out in our licence referred to above.

Other than as permitted in any relevant BMJ Author's Self Archiving Policies, I confirm this Work has not been accepted for publication elsewhere, is not being considered for publication elsewhere and does not duplicate material already published. I confirm all authors consent to publication of this Work and authorise the granting of this licence.

For peer review only

TITLE PAGE

Design considerations for the migration from paper to screen media in current health education for older adults: A scoping review

Larissa Taveira Ferraz[1]

Anna Julia Tavares Santos[2]

Lorena Jorge Lorenzi[3]

David M. Frohlich[1]

Elizabeth A. Barley[4]

Paula Costa Castro[2]

1. Department of Music and Media, University of Surrey, Guildford, United Kingdom.
2. Department of Gerontology, Federal University of São Carlos, São Carlos, Brazil.
3. Programa de Pós-graduação Interunidades em Bioengenharia, University of São Paulo, São Carlos, Brazil.
4. School of Health Sciences, University of Surrey, Guildford, United Kingdom

Corresponding author:
Larissa Taveira Ferraz

l.taveiraferraz@surrey.ac.uk

Digital World Research Centre, Room 15AP01, University of Surrey, Guildford GU2 7XH, UK

Keywords: Health education, Older people, Paper-based media, Screen-based media, Review.

Word count: 7182

For peer review only

Design considerations for the migration from paper to screen media in current health education for older adults: A scoping review

ABSTRACT

Objective: To map the current use of paper-based and/or screen-based media for health education aimed at older people. **Methods**: A scoping review was carried out following the PRISMA-ScR. The search was carried out in seven databases, with studies available in the last 10 years, in English, Portuguese, Italian or Spanish, in addition to a Google Scholar search to check the grey literature. The terms used in the search strategy were older adults, health education, paper and screen-based media, preferences, intervention and other related terms. Studies were included that carried out health education interventions for older individuals using paper and/or screen-based media and that described barriers and/or facilitators of the use of these media. The selection of studies was carried out by two reviewers, in five stages. **Results:** The review included 21 studies that carried out health education interventions with different purposes, the main ones being promotion of physical activity, hypertension prevention and psychological health. All 21 interventions involved screen-based media on computers, tablets, smartphones and laptops, while only 4 involved paper-based media such as booklets, brochures, diaries, flyers and drawings. This appears to reflect a transition from paper to screen media for health education for an older population, in research if not practice. However, analysis of facilitators and barriers to use of both media revealed 10 design factors that could improve or reduce use, and complementarity in their application to each media type. For example, screen media could have multimedia Content, additional Functionality and interactivity through good Interaction Design, but have low Accessiblity and require additional Learning due to complex Interface Design. Conversely, paper media had static Content and low Functionality but high Accessibility, Availability and a low Learning overhead. Conclusions: We recommend improved screen media design, continued use of paper, and the possible combination of both media through the new augmented paper technology.

Registration number: Open Science Framework (DOI: DOI 10.17605/OSF.IO/GKEAH)

Strengths and limitations of this study

- It is the first review that mapped the differences in the use of paper-based and screen-based media for health education for older people.
- The studies were searched in seven databases, in addition to a grey literature search.

For peer review only

- The studies were verified in four languages, being Portuguese, English, Spanish and Italian.
- A limitation of the review was to have as an inclusion criterion that the studies should have a mean age equal to 60 years or older.

INTRODUCTION

The population of older adults aged 60 and overreached about 14% of the world's population in the year 2020, which means 1 in 7 people. In the year 1950, this percentage was 8% of the population.[1] Population ageing has been triggered by a series of factors, such as a decrease in the fertility rate and an increase in life expectancy.[2] The projection for 2050 is that adults over 60 will reach 22% of the world's population, that is, approximately 1 in 5 people.[1] This increase is also leading to more people living with chronic health conditions and a need to improve quality of later life through what is called healthy ageing.

The document entitled "Decade of Healthy Ageing", by the World Health Organization (WHO), proposes a plan to improve the health of the older adult population between 2021 and 2030.[3] It emphasises the importance of health conditions being managed efficiently with health promotion and disease prevention practices.[3] A key part of this strategy is the provision of effective health communication, through media that are disseminated both on paper and screen. According to WHO, health communication can be defined as "the use of communication strategies (eg, interpersonal, digital and other media) to inform and influence decisions and actions to improve health". [4]

Paper-based media are established formats for health education provided by professionals due to their widespread accessibility.[5] Such media were, and still are, widely used in health practices in the form of booklets, leaflets, posters and manuals.[6] In the literature, there are some studies that present recommendations and guidelines for their use, such as the one carried out by Hoffmann and Worrall (2004) [5], they emphasise the importance of these materials being: objective and updated; easy to understand for everyone; using table of contents, subheadings, bulleted lists, and abstracts; organised, with differentiation between uppercase and lowercase letters and use of bold only for emphasis; with the use of images or drawings only to aid understanding; and with the use of interaction resources, for example a short questionnaire.

Increasingly, health education materials are being disseminated through applications and websites on screen-based devices such as smartphones and computers.[7] Many of these

For peer review only

health education services are targeted at older adults, because they are disproportionately affected by illness and are likely to benefit most from information about healthy ageing practices. It should also be noted that, along with technological advances, there are other situations that enhance the use of digital media, such as the advent of the COVID-19 pandemic and the necessary social distancing.[8] Recommendations on the use and design of screen-based health information are also available in the literature. For example, the study by Zhao, Zhao and Song (2022) [9] explores the information seeking behaviour of older adults for health information and reveals that in addition to personal barriers, there are barriers in technology, such as unsatisfactory interactivity and usability, inadequate font sizes, dense texts, lack of visual elements and confusing layouts.

Current studies, including reviews, of the use of paper or screen formats for health information are carried out separately and seldom compared with each other. Furthermore, they address a wide range of ages in the audiences involved. The aim of this review is to map the use of paper and screen based media in current health education practices targeted at older people aged 60 or over. This should allow the identification of the barriers and facilitators in the use of each media for this population, comparing the use of these two types of media for health education.

METHODS AND ANALYSIS

This review was developed according to the guidelines of the *Preferred Reporting Items of Systematic Reviews and Meta-analyses for Scoping Reviews* (PRISMA-ScR) [10] and the recommendations of the Joanna Briggs Institute Revisions Manual (JBI). [11] The protocol of the review with methodological details is published.[12] The research questions were:

1. What types of paper-based and screen-based media are used in current forms of health education and how are they used for health education of older adults?
2. What are the barriers and facilitators to using paper-based and screen-based media for health information of older adults?
3. What are the differences between the use of paper-based and screen-based media for health education?

Eligibility, inclusion and exclusion criteria

For peer review only

Scientific articles that had as their objective or central theme a health education intervention carried out with paper and/or screen-based media for health promotion or prevention of older individuals were considered eligible. Studies were included that carried out or described health education interventions for health promotion or prevention of older individuals using paper and/or screen-based media and that described barriers and/or facilitators of the use of these media. The studies included should have a sample of subjects with a mean age of 60 years or older and be published in English, Portuguese, Spanish or Italian, from 2012 until the search date. In addition, grey literature studies were included through an additional search in the Google Scholar.

Studies that were not about health promotion or prevention, such as interventions on treating or caring for pre-existing health conditions were not included. In addition, studies that did not present barriers or facilitators perceived by users in relation to the media were excluded. Political documents, technical reports and studies whose full texts could not be obtained, even after attempts to contact the authors, were excluded.

Search Strategy

A search strategy was developed based on preliminary searches in databases, using the terms *older adults, health education, paper-based media, screen-based media, experience, intervention*, in addition to other related terms. The search strategy was adapted using Boolean operators and Mesh terms according to each database. The search strategy used in Scopus databases was:

(“older person“ OR elder* OR “older adults” OR “elderly population” OR “older people” OR ageing OR aging OR “older population” OR geriatric OR “healthy ageing” OR “successful aging”) AND (“paper based media” OR “screen based media” OR website* OR platform* OR virtual OR online OR multimodal OR multimedia OR “reading media” OR “digital-based reading” OR “paper based reading” OR flyer OR “media advertisement” OR “print media” OR app OR apps OR tablet* OR smartphone* OR m-health* OR e-health* OR “patient information leaflets”) AND (“health education“ OR “health information” OR “health communication” OR “health promotion”) AND (preference* OR characteristic* OR experience* OR attribute* OR perception* OR development OR barrier* OR facilitator* OR opportunities OR problem* OR recommendations) AND (trial OR intervention)

Searches were performed in seven databases: Scopus (Elsevier), Web of Science (Clarivate), Medline, Embase (Elsevier), Cinahl (EBSCO), The ACM Guide to Computing Literature, Psyinfo (APA) databases. Studies published in Portuguese, English, Spanish and Italian, from 2012 to the date of the search in June 2022, were searched. An additional search

For peer review only

on Google Scholar was performed to verify 150 studies according to Google's relevance algorithm in the year 2022. Later, in April 2023, after guidance from the protocol reviewer that suggested the search should include the first 300 studies, based on the paper by Haddaway NR (2015),[13] the expansion of the grey literature was carried out, updating the search for April 2023, until the first 300 studies from Google Scholar were identified.

Study/Source of evidence selection

The selection of studies was carried out in five stages, starting with the search in the databases in which the identified studies were extracted into the Excel program and duplicates were removed. The next step was a pilot test in which two reviewers checked 25 random studies found in the search according to the eligibility criteria, obtaining a 92% agreement rate.

The third step was the reading of abstracts, titles and keywords of all studies by two reviewers independently following the eligibility criteria. And the fourth step was the reading of the full texts of the studies accepted in the previous step by the reviewers independently according to the inclusion criteria. In both phases, a third reviewer resolved disagreements. The last step was the additional strategy, which was the reading of the first 300 studies found in Google Scholar according to the relevance algorithm.

Data extraction

Data from the studies included in the review were extracted into a table developed and checked by the authors in three studies. The extracted data contained information about the studies such as authors' names, title, year of publication, country of origin and methods used, information about the sample such as size, gender and mean age, characteristics of the intervention such as duration and frequency, health condition verified, paper media used, screen media used, barriers to the use of paper and/or screen media and facilitators in the use of paper and/or screen media. The extraction table was described in the protocol for this scoping review, however the topic "Is the health education aimed at the caregiver?' was withdrawn. This topic was removed from the extraction table, considering that throughout the study it was identified that the papers included were mostly focused on the participants 60+ and not caregivers.[13]

Data analysis and presentation

The data extracted from the included studies were presented in a narrative form and through tables and the PRISMA flowchart. Quantitative data were analysed using relative and

For peer review only

absolute frequency, mean and standard deviation. Qualitative data about the barriers and facilitators in the use of paper and/or screen-based media were analysed using Bardin's content analysis.[14] Barriers and facilitators were grouped into categories according to their similarity.

RESULTS

The databases searched resulted in 1,392 studies, as shown in the flow diagram in figure 1. Duplicate studies were removed which resulted in 1,080 articles found. All the studies found were screened by the two reviewers, and according to the eligibility criteria, a total of 134 articles were sent to the reading step of the full texts. In the selection of studies by the inclusion criteria the total number of included studies was 15 and 119 were excluded as described in the flowchart (Figure 1). In the additional strategy, which was the reading of the 300 studies found in Google Scholar in order of relevance, it was identified that 21 were duplicates that had already been found in the search of the databases. Thus, in order to have 300 studies without duplicates, 321 studies were screened. As a result, 95 studies were selected for full reading and, subsequently, 6 were included based on the same inclusion criteria applied to full texts. Therefore, the main search included 15 studies and the Google Scholar search 6 studies, this review then included a total of 21 studies.

Figure 1: PRISMA flow diagram of the studies included in the review that presents screen-based and/or paper-based media for health education.

(FIGURE 1)

Description: Reason 1: it is a protocol, literature review or it has only an abstract available; Reason 2: it does not present the mean age or the mean age is less than 60 years; Reason 3: it does not have participants; Reason 4: it does not have a health education or the health education is not for promotion or prevention; Reason 5: health education does not use paper or screen-based media; Reason 6: it does not describe the health education; Reason 7: it does not present barriers or facilitators perceived by users in relation to the media used.

Source: developed by the authors.

For peer review only

Characterization of the studies

Descriptive characteristics of the included studies (n = 21), such as year of publication, country where it was carried out, year of data collection, methodology, intervention, sample size and average age of participants are described in Table 1.

Table 1 – Descriptive characteristics of the included studies

Variables	n	%
Year of publication		
2012	1	4.76
2013	1	4.76
2014	2	9.52
2015	2	9.52
2016	2	9.52
2017	2	9.52
2018	5	23.81
2019	2	9.52
2021	3	14.29
2022	1	4.76
Location - Country		
United States	5	23.81
Australia	3	14.29
Netherlands	3	14.29
New Zealand	2	9.52
South Korea	2	9.52
Belgium	1	4.76
Malaysia	1	4.76
Germany	1	4.76
Taiwan	1	4.76
International (several countries)	2	9.52
Year of data collection		
2007-2009	1	4.76
2010-2011	1	4.76
2012	2	9.52
2014-2015	1	4.76
2015	1	4.76
2016	1	4.76
2017	1	4.76
2017-2018	1	4.76
2020	1	4.76
2020-2021	1	4.76
NR	10	47.62
Methodology		
Longitudinal	17	80.95

For peer review only

Cross-sectional	4	19.05
Intervention		
In-person	11	52.38
Online	2	9.52
In-person and online	8	38.10
Sample size		
05-30	6	28.57
31-60	7	33.33
84	1	4.76
121-150	4	19.05
204	1	4.76
218	1	4.76
703	1	4.76
Average age of participants		
60-64 years	5	23.81
65-69 years	7	33.33
70-74 years	4	19.05
75-79 years	3	14.29
Unknown	2	9.52

NR: Not reported
Source: developed by the authors.

Regarding the year of publication described in table 1, approximately 61.9% of the articles were published in 2017, 2018, 2019, 2021 or 2022. Respectively, the United States, Australia, Netherlands, New Zealand and South Korea were the countries that most originated studies in this review. The year of data collection in the studies showed great variability, with the oldest year being 2007 and the most recent year being 2021. The majority of studies were carried out longitudinally (80.95%).

The interventions were mostly face-to-face (52.38%) and had a minimum duration of 30 minutes and a maximum duration of 6 months. The studies' sample ranged from 5 to 703 participants, however, mostly had 31 to 60 participants (33.33%) or 5 to 30 participants (28.57%) and most of the studies had an average age between 65 to 69 years old (33.33%) or 60 to 64 years old (23.81%). Two studies (9.52%) did not report mean age, but did not have adults (aged 18 to 59) in the sample. The mean total age of participants across all included studies was 68.51 (±5.06) years. Most studies (90.48%) were performed with individuals of both genders and the other studies did not report data on gender. In addition, 47.62% of the studies did not have adults (aged 18 to 59) in the sample, 47.62% of the studies had adults, and one study did not report this information, but the mean age was over 60 years.

Health education themes and types of media found in the studies

For peer review only

In response to the first research question of this review about the *formats* of health information used and for what purposes, Table 2 presents all the studies included in this scoping review, with their authors, year of publication, central theme of the health education and what type of digital/ paper media format was used.

The central theme of health education had great variability among the included studies, however it was identified that the themes that most appeared were physical activity promotion (14.29%), psychological health (14.29%) and hypertension prevention and care (9.52%).

Among the screen-based media formats used for health education in the studies, the following media were identified: website (61.90%), app (23.81%), email (9.52%) and animations on Microsoft PowerPoint (4.76%). These media were used on the devices: computers (52.38%), tablets (28.57%), smartphones (19.05%) and laptops (4.76%). Some studies used more than one media or device. Regarding the paper-based media formats used for health education in the studies, it was identified that they were booklets (A4 Paper), brochures, handouts, diaries, flyers, drawings and print-delivered information (without specification).

A major finding of the review was the imbalance of studies on paper and screen interventions. All 21 articles included in this study featured screen-based media, and among these, only 4 also reported the contrasting use of paper-based media. No studies were found that reported on the use of paper-based interventions alone.

Table 2: Studies included in the review, health education provided and types of paper-based and/or screen-based media used.

Article title	Authors, Year	Central theme of the health education	Screen-based Media Format	Paper-based Media format
A Digital Human for Delivering a Remote Loneliness and Stress Intervention to At-Risk Younger and Older Adults During the COVID-19 Pandemic: Randomized Pilot Trial [15]	Loveys et al., 2021	Mental health and well-being	Computer, tablet or smartphone (website)	NA (Not applicable)
A Multi-faceted Approach to Promote Comprehension of Online Health Information Among Older Adults[16]	Chin et al, 2018	Self-care regarding hypertension	Computer	NA
Acceptability of a guided self-help Internet intervention for family caregivers: Mastery over dementia [17]	Pot; Blom; Willemse, 2015	Psychological distress of family caregivers of people with dementia	(Website)	NA

For peer review only

Assessing the Impact of a Game-Centered Mobile App on Community-Dwelling Older Adults' Health Activation [18]	Crandall; Shake; Ziegler, 2019	Risk of falls and osteoarthritis	Tablet (App)	NA
Co-design of an evidence-based health education diabetes foot app to prevent serious foot complications: a feasibility study[19]	Ogrin et al, 2018	Foot health for people with diabetes	Smartphone (app)	NA
Development and assessment of a web-based intervention for educating older people on strategies promoting healthy cognition[20]	Vanoh et al, 2018	Precautionary strategies for mild cognitive impairment	Computer (Website)	NA
Development and Evaluation of a Computerized Multimedia Approach to Educate Older Adults about Safe Medication[21]	Im & Park, 2014	Safe medication	Computer (Website)	NA
Development and Validation of an Interactive Internet Platform for Older People: The Healthy Ageing Through Internet Counselling in the Elderly Study[22]	Jongstra et al, 2017	Improving the cardiovascular risk profile of old people with high cardiovascular risk	Computer (Website)	NA
Development of a communication intervention for older adults with limited health literacy: Photo stories to support doctor–patient communication[23]	Koops et al., 2016	Doctor-patient communication and communicative health literacy	(Website)	Booklet (A4 Paper)
Differences in the use and appreciation of a web-based or printed computer-tailored physical activity intervention for people aged over 50 years[24]	Peels et al, 2013	Physical activity promotion	Computer (Website; email)	Print-delivered information, brochures and handouts
Efficacy of a web-based, center-based or combined physical activity intervention among older adults[25]	Mouton & Cloes, 2015	Physical activity	(Website; email)	NA
Evaluation of the usability and acceptability of the InnoWell platform as rated by older adults: Survey study [26]	Lamonica et al 2021	Biopsychosocial domains	Tablet, computer, laptop, smartphone (Website)	NA
Improving older adults'e-health literacy through computer training using NIH online resources[27]	Xie; Bo, 2012	Improve the ability of seniors to search, find and understand health information online	Computer (website)	NA

For peer review only

Mobile App Use for Insomnia Self-Management in Urban Community-Dwelling Older Korean Adults: Retrospective Intervention Study[28]	Chung et al, 2018	Sleep education	Smartphone (app)	NA
Self-efficacy, self-regulation, and physical activity behavior in type 2 diabetes[29]	Olson, 2014	Metabolic Health Education	(Website)	NA
The Application of Virtual Reality in Patient Education[30]	Pandrangi et al, 2019	Health education - health status	(Website)	Flyers, drawings and brochures
The GezelschApp A Dutch Mobile Application to Reduce Social Isolation and Loneliness[31]	Jansen-Kosterink, Varenbrink & Naafs, 2018	Social isolation and loneliness among old people	Computer and tablet (App)	NA
The Impact of 3-D Models versus Animations on Perceptions of Osteoporosis and Treatment Motivation: A Randomised Trial[32]	Jones et al, 2017	Information about osteoporosis	Tablet (animations on Microsoft PowerPoint)	NA
The influence of robot-assisted learning system on health literacy and learning perception[33]	Wei et al, 2021	Hypertension prevention for older adults	Tablet and computer	NA
Web-based oral health promotion program for older adults: Development and preliminary evaluation[34]	Marino et al, 2016	Oral health promotion	Computer (Website)	NA
Web-based versus print-based physical activity intervention for community-dwelling older adults: crossover randomized trial[35]	Pischke et al., 2022	Promoting a physically active (PA) lifestyle	(Website and app)	Brochures and diary

NA: Not applicable.
Source: developed by the authors.

For peer review only

Categorization of barriers and facilitators

In response to the second research question about the barriers and facilitators to use, it was identified that the vast majority were about screen-based media. Barriers were considered to be difficulties that older people had when using screen or paper media, factors that hindered the use or the lack of features. Facilitators, on the other hand, were factors that contributed or motivated individuals to use the media. Thus, they were grouped according to their similarity into 10 categories, described below:

- Accessibility - the diversity of users able to access the media system.
- Availability - the availability to use the media system.

- Content design - the design of the media content itself.
- Functionality - what the media system does.
- Interaction design - design of the sequential organisation of the interaction with the media system.
- Interface design - design of the input and output interfaces.
- Learning - the ease of learning to use the media system.
- Privacy - privacy of personal data in the media system.
- Social interaction - the support of interaction with other people around the media system.
- Usability - the ease of using the media system.

The topics defined for the categorisation of barriers and facilitators seem to be properties of the design of media systems in general that can have both negative (barriers) and positive (facilitators) effects. They are different sides to the same coin because if done well they improve health education, but if done badly they reduce it. The categories found in barriers and facilitators related to screen-based and paper-based media are described in Table 3. The table describes the number of studies that identified each category, with some studies finding more than one type of category as barriers and/or facilitators. In the following sub-sections we step through the cited barriers and facilitators for each medium in turn.

Table 3 - Barriers and facilitators found in the included studies.

Topics	Screen-Based Media		Paper-Based Media	
	**Barriers**	**Facilitators**	**Barriers**	**Facilitators**
**Accessibility**	5	3	--	--
**Availability**	1	3	--	2
**Content design**	4	8	--	1
**Functionality**	3	6	2	--
**Interaction design**	2	4	--	--
**Interface design**	2	4	--	--
**Learning**	6	4	--	--
**Privacy**	1	--	--	--
**Social interaction**	1	2	--	--

For peer review only

**Usability**	3	6	--	--

Source: developed by the authors.

Barriers of screen-based media

The main categories that appeared as barriers to the use of screen-based media were Learning (e.g. lack of computer training, lack of instructional videos, the participants wanted clear guidelines, older adults required technology training to be completed in-person), Accessibility (e.g. would prefer a larger screen using a tablet or laptop, prefer hard copies of the information, "a sad thing is you can't touch her or talking to a real person"), Content design (e.g. strange contents, technical terms, information wasn't as up to date, participants wanted varied levels of information), Functionality (e.g. lack of reward, some technical improvements are needed like the function to delete messages, defining a goal was difficult for the participants, an icon to facilitate printing would be useful) and Usability (e.g. the assignments were not direct and required big effort, passwords provided to login for the first time were too difficult). However, the following barriers were also identified: Availability (e.g. computer availability and computer reserves), Interaction design (e.g. aspects participants felt could be improved were the human likeness of it interaction like behaviours and voice and aspects of the conversation design), Interface design (e.g. videos were not easy to see because they had small size, small font size, lack of attractive photos and project colours), Privacy (e.g. participants did not post on the discussion board due to privacy concerns), Social interaction (e.g. limited app interaction with participants).

Facilitators of screen-based media

The main categories that appeared as facilitators for the use of screen-based media were Content design (e.g. information with revised content by researchers and experts helped them to understand the content, easy-to-understand text structures, bulleted lists, and phrases, animation based on a life story of an older adult character contributed to enhancing the self-efficacy of the participants), Usability (e.g. operating the program just by clicking the mouse was easy, it was simple and easy to navigate on a mobile phone, easy to use interface, most participants found these sites easy or very easy to use), Functionality (e.g. participants really liked the function to be able to make new friends and send and receive messages, participants mentioned videos as the most useful components, feedback was helpful), Accessibility (e.g. the app was appropriate for people with a low level of education, touch screen facilitates the use by old adults) and Interaction design (e.g. participants appreciated the interactive features

For peer review only

of the platform like the interactive videos, interactive questions help with problem-solving, participants liked the interpersonal skills). However, the following facilitators were also identified: Availability (e.g. information on the smartphone can be seen at any time quickly, permanent access to the website), Interface design (e.g., the animations on the tablet allowed more of the information to be visualised, participants liked the appearance, cross-referenced a number of the images and statements to various sections), Learning (e.g. participants reported that they easily learned how to use the application,, they also enjoyed and felt comfortable with self-learning), Social interaction (e.g. many participants expressed how much they enjoyed the social aspect of the app, participants considered the app a pleasant and safe way to find new friends).

Barriers of the use of paper-based media

Regarding the barriers related to paper-based media, the study by Pischke et al (2022) [35] found Functionality to be a barrier as the printed version seemed to be impractical regarding physical activity. Pandrangi et al (2019) [30] also identified Functionality as a barrier since participants perceived paper as a traditional method and considered virtual reality more useful.

Facilitators of the use of paper-based media

In the study by Koops et al. (2016) [23] it was identified that the paper was a facilitator in relation to Availability, as the participants reported that they could take it anywhere and Content design, as they liked that the stories were similar to real life and that it was easier for them to understand. Peels et al. (2013) [24] found availability to be a facilitator since the participants had more intentions to keep the information and to use the concepts learned in the future.

Differences between the use of paper-based and screen-based media for health education

Regarding the third research question about the differences between the use of paper-based and screen-based media, this was difficult to answer because of the small number of direct comparisons made. However, three particular barrier/facilitator categories appeared to

For peer review only

relate to the contrasting properties of paper and screen, as expressed in the four studies using paper and screen materials. These were Availability, Functionality and Learning.

Availability appeared in two studies as facilitators concerning the use of paper media due to the possibility of having the paper in hand and being able to use it at any time. For the use of screen-based media, there is a need for more effort, for example, when having to turn on a computer, log in to a website, download an application, etc., in order to be able to start using this media. [23, 24]

Considering the use of paper-based media, two other studies identified Functionality as a barrier to their use, due to the fact that this is limited compared to screen media, in which there is the possibility of having different functions, such as having feedback, interactive videos, setting goals and being able to make new friends. [30, 35]

In addition, the Learning category is related to the difference in the use of paper and screen due to the older audience having individuals who have difficulty using screen media, as they have no affinity with it and need training to use them. [35, 25] Thus, for individuals with difficulty in relation to the use of screen media, the use of paper is preferable, as seen in the study by Pischke et al. (2022). [35]

DISCUSSION

The review identified 21 studies that carried out health education interventions to promote ways of staying healthy longer to an ageing population subject to more chronic health conditions, the main subjects in the interventions were related to promotion of physical activity, hypertension prevention and psychological health. This was reflected in the health education subjects of the papers . These were directed to issues such as metabolic health, sleep, healthy eating, physical activity and cognition, and focused on the development and maintenance of autonomy and independence, enabling healthy ageing, according to the WHO definition. [3]

The interventions were done through the use of screen-based media such as websites, apps and emails, on desktop computers, tablets, smartphones and laptops, and paper-based media such as booklets, brochures, diaries, flyers and drawings. We found that the barriers and facilitators to the use of these media were essentially design properties that could improve media use if done well or reduce use if done badly. This led us to propose a taxonomy of 10 design features and considerations which apply to both media, somewhat unequally. The main design considerations for the use of screen-based media were related to Content design, Functionality, Usability, Accessibility and Learning. The main design considerations for the

For peer review only

use of paper-based media, were Availability and Content design, but such media were often considered traditional because they did not have extra functionality.

We can see from the diversity of paper and screen materials in the studies that these terms each refer to quite different formats. In fact, they gloss these differences and do not really do justice to the different interactions people could have with each format. This may be one reason for the ambiguity of findings in such comparisons in the past, and suggests greater attention to *particular* paper and screen formats in future research. For example, screen information was presented on four physical devices in the above studies, including desktop and laptop computer, tablet and smartphone. Sometimes this information was on a website or presented in a more interactive app, leading to a matrix of 8 possibilities across devices with different user experiences. Likewise, paper information was presented on single page leaflets, fliers and handouts, as well as multi-page booklets or diaries. Each of these has different affordances for reading, finding, handling and capturing information, and are not equivalent user experiences. Further work is required to understand the subtleties of design, interaction and experience will all these 'paper' and 'screen' formats, since the current papers do not go into sufficient detail.

Regarding the types of digital media used for health education, the World Health Organization has a 'Global Strategy on Digital Health 2020-2025' [36] with the purpose of strengthening health systems through digital health technology and this will engage patients to achieve the vision of health and well-being for all. This strategy signals that practices for public awareness of digital health must be addressed, this includes health literacy and patient engagement. [36] Our findings clearly reveal a noticeable trend towards digitization and a preference for screen-based media in the literature. When analysing the review data from the last decade, we observed a significant increase in the number of studies and publications focusing on screen-based media across all years, as depicted both in Tables 1 and 2. Our study found barriers and facilitators that, if properly addressed, will make new digital health strategies more user friendly for older people, which will improve the engagement of this group in health education interventions and consequently improve health literacy. Key amongst these were the design of *functionality* and *content*, which were shown to have a great impact, both positive and negative, on interventions.

Based on the findings of our review, the discussion in the literature emphasises the significance of interface design and screen media usability. Morey et al. (2019) [37] identified issues with an excess of icons and low contrast, highlighting participant concerns. Vanoh et al.'s (2018)[20] study underscored that older users prefer visually appealing media featuring

For peer review only

attractive photos and colours, while Jongstra et al. (2017)[22] revealed their need for different functionalities. Hence, it is imperative to incorporate diverse functionalities tailored to the requirements of the older population, coupled with an engaging design characterised by colour contrast and intuitiveness to enhance usability.

The adoption of a user-centred design approach is crucial to meet the unique needs of older audiences. Co-design sessions, as suggested by Leme (2014)[38], serve as an effective strategy for involving older individuals in the creation and design processes of new tools across various media. In this way, participatory design enables the creation of intuitive media, which, consequently, older individuals will find easy to use. Moreover, it was identified in the included studies that one of the main barriers was the difficulty of learning these screen media. Therefore, it is crucial that public policies are developed and improved for the digital inclusion of older people, such as programs that teach these individuals how to use these media, so that they can enjoy all the benefits of digital technologies. The study by Chung et al. (2018)[28], which aims to analyse the usability of a sleep education app for older Koreans, presents as a perceived facilitator by users the ease with which they learned how to use the application.

Regarding screen media use, learning to use the media was one of the most prevalent barriers. According to De Paiva and Alves (2018) [39], the current population of older users accompanied the emergence of the internet, the printer and the computer. Nowadays, with a technological generation, games with different levels of proficiency and the emergence of artificial intelligence, the older population finds it difficult to adapt.

In one of the studies of this review, by Pischke et al. (2022)[35], that compared the acceptance and effectiveness of two interventions for physical activity (PA) promotion for older adults through websites, apps, brochures and diaries, they found individuals who have no affinity with screen media, preferring paper. In another study found in this review, by Peels et al. (2013)[24], based on a perception considered an Usability barrier, that reports "The assignments integration into the web-based tailored advice were not direct and required big effort to participate", it is notable that not being intuitive leads to a greater amount of effort. Thus, older people have greater difficulty with screen-based media and require more training. This training requirement is urgent at the current moment, given that the current young population, who are the future older people, are already growing up with skills and will likely have an easier time using them.[24]

Additionally, functionality is identified as a barrier in the use of e-health for older adults, as noted in Wilson's (2021)[40] scoping review. Conversely, ease of use emerged as a

For peer review only

facilitator, as observed in both Alruwaili's (2023)[41] and Wilson's (2021)[40] studies, aligning with our usability review category.

Our study's results align with concerns mentioned in Olson's (2014)[29] research, particularly regarding privacy, which is also identified as a barrier in related studies such as Alruwaili's, 2023[41] systematic review and Wilson's, 2021[40] study. Privacy and data security apprehensions are recognised as obstacles to digital health interventions among older adults. Therefore, it is essential to create public policies that ensure the privacy and security of users' data when using screen-based media in health education interventions. It is also important that policies regarding screen media are presented to the user at the beginning of the interaction with the media, as this will inform and ensure safety for users.

Digital engagement carries the danger and opportunity of data capture and user profiling. The Global Strategy on Digital Health 2020-2025[36] emphasises that health data is sensitive and personal, it requires a high standard of security and protection. Lack of trust and confidence about personal health data can discourage people from fully participating in health education interventions. WHO mentions the need for a strong legal and regulatory foundation to protect privacy, integrity, confidentiality and deal with data cyber security. It is also important to maintain transparency and communicate effectively about data security strategies to users. [36]

Regarding the use of paper media for health education, according to the findings of this review, it is clear that these media are still very much alive today, as revealed in the study by Koops et al. (2016) [23], that used a website and a booklet to development a doctor-patient communication for older adults with limited health literacy. In this study, a booklet with photographic stories that aided in the communication between doctor and patient was considered to have good availability, as it could be given out and used anywhere. This is likely to reflect a pervasive practice of displaying posters and giving out leaflets in GP surgeries and hospitals across the globe, regardless of their lack of representation in this research review. Indeed, this is mentioned in the wider literature. This is supported by wider literature, such as a study by Maskell, McDonald and Paudyal (2018)[42], which found that more than three-quarters of patients noticed printed materials in the waiting rooms of general clinics in England, and a large proportion read them. Hence, paper-based media are already widely accepted and available as part of modern health education practices.

Regarding the international reach of these studies we note that they come largely from high income countries as shown in Table 1. There is a lack of research and representation from low and middle income countries, especially those with vulnerable populations, and large

For peer review only

countries with vast and remote regions facing challenges in accessing healthcare services, as shown in Table 1. For populations with limited education and financial vulnerability, paper-based materials appear more accessible due to their traditional usability and cost-effectiveness. However, for individuals who are actually illiterate, screen readers and audio content, for instance, offer a level of accessibility that paper cannot match. These functionalities are facilitators found in screen-based media seen in this review, as videos mentioned as the most useful components in the study of Olson (2014)[29]. Moreover, advancements in satellite connection technologies are now integrating previously isolated areas into the global network, potentially granting access to health information for a substantial number of people who currently lack access to up-to-date pamphlets and leaflets. This integration and interactivity brought about by screen-based media could also enable these individuals to connect with health teams and professionals in more densely populated areas. This, in turn, could lead to more personalised and humanised health education guidelines and interventions through technology. Overall, these developments hold promise in bridging the healthcare information gap and fostering improved health outcomes for underserved communities.

Given the advantages and disadvantages of paper and screen-based information emerging from this review, it may be valuable to combine them in future health campaigns, perhaps at a new level of granularity. This can be done through *augmented paper* technology which supports the reading of printed hotlinks on a nearby smartphone.[43] This results in a paper-and-screen reading experience in which users move back and forth between media which have been carefully designed to complement each other. For example, a printed travel guide can play interviews with local characters, video walks through the landscape, and trigger phone calls to organisations or show updated timetable information.[44] The user in this scenario does not have to know how to use the web to access the digital information. They simply take a picture of the printed page, speak its page number, turn the page or move a bookmark to access the links.[45] A similar approach could be taken to the design of augmented patient information leaflets, posters and cards, which could play multimedia illustrations, spoken translations or instructions, and call local clinics or medical services for more advice.[46]

This review is limited to studies reporting the use of participants with a mean age equal to 60 years or older. Thus, studies that had not reported the mean age or age range of participants were excluded, but it may be that some would have been included if they had reported this information. The strengths of this review are: this is the first study that identifies and compares digital and paper media for health education aimed at older people, as well as design considerations related to their use; studies were searched for in seven databases and in

For peer review only

the grey literature; studies were searched for in four languages, namely Portuguese, English, Spanish and Italian; systematic methods were used for the selection of studies e.g. all titles, abstracts and full texts were screened by two reviewers independently. Another limitation we encountered was the scarcity of studies exclusively utilising paper-based media, as none were solely dedicated for this form, and only 4 out of 21 studies found contrasted paper with screen media. Following the completed review, we identified 'printed material' as a potential descriptor that other researchers can use. While this trend might indicate the increasing use of screen media, it limits the enhancement of comparisons between the barriers and facilitators of both.

CONCLUSION

A transition or migration appears to be taking place in the provision of health information from paper to screen. In this review we have examined recent literature on the design and use of such information for older people across these formats and found an over-representation of research on screen interventions. Although this suggests that the future lies in greater digital engagement, a careful reading of 21 papers shows many barriers to the use of screen-based information and corresponding facilitators to the use of paper information for health, especially for an older population and broader international context. We therefore recommend greater attention to the barriers of screen-based information across different devices and services, continued use of paper, and the possible combination of both media through new augmented paper technology.

For peer review only

ETHICS AND DISSEMINATION

Ethical approval is not applicable to this scoping review, it only gathered information from published literature. The results will be disseminated through presentations at significant scientific events and published in journals in the area.

Author Contributions: All authors have made substantial contributions to the development of the manuscript. LTF and AJTS conceptualised the research question and prepared the first draft of the manuscript. LTF, AJTS, LJL, PCC, DMF, EAB contributed to the refining of the study design and guided the review development. Also, LTF, AJTS, and LJL analysed the results and PCC, DMF, EAB contributed to the discussion. Also LTF, AJTS, LJL, PCC, DMF, EAB contributed to the writing, and revising this review. DMF assisted in the revision of the English.

Competing interests: The authors declare no conflict of interest.

Funding: LTF received funding from the University of Surrey Doctoral College (Breaking Barriers Studentship Award), AJTS received funding from São Paulo Research Foundation (FAPESP), N° 2022/08918-6 to the development of this review and LJL received funding from Coordination for the Improvement of Higher Education Personnel (CAPES) - Financial Code 001.

Data sharing statement: Data from this review are available in this manuscript and in the previously published protocol. As it is a scoping review, the sharing of other data is not applicable.

Patient and Public Involvement statement: None.

Acknowledgements: We would like to acknowledge University of Surrey Doctoral College, São Paulo Research Foundation (FAPESP), Coordination for the Improvement of Higher Education Personnel (CAPES) for funding.

For peer review only

REFERENCES:

1. United Nations, Department of Economic and Social Affairs, Population Division (2022). World Population Prospects: The 2022 Revision. 2022. Available from: https://population.un.org/dataportal/.
2. United Nations Department of Economic and Social Affairs, Population Division (2022). World Population Prospects 2022: Summary of Results. 2022b. UN DESA/POP/2022/TR/NO. 3.
3. World Health Organization. Decade of healthy ageing: baseline report. Geneva, 2020.
4. World Health Organization. Health promotion glossary of terms 2021. 2021
5. Hoffmann, T., & Worrall, L. (2004). Designing effective written health education materials: considerations for health professionals. Disability and rehabilitation, 26(19), 1166-1173.
6. Sá, G. G. D. M., Silva, F. L., Santos, A. M. R. D., Noleto, J. D. S., Gouveia, M. T. D. O., & Nogueira, L. T. (2019). Technologies that promote health education for the community elderly: integrative review. Revista latino-americana de enfermagem, 27.
7. Kampmeijer, R., Pavlova, M., Tambor, M., Golinowska, S., & Groot, W. (2016). The use of e-health and m-health tools in health promotion and primary prevention among older adults: a systematic literature review. BMC Health Services Research, 16(5), 467-479.
8. Celuppi IC, Lima GS, Rossi E, et al. An analysis of the development of digital health technologies to fight COVID-19 in Brazil and the world. Cad Saude Publica 2021;37(3):e00243220. Doi: 10.1590/0102-311X00243220.
9. Zhao YC, Zhao M, Song S. Online Health Information Seeking Behaviors Among Older Adults: Systematic Scoping Review. Journal of medical Internet research 2022; 24 (2): e34790. DOI: 10.2196/34790
10. Tricco AC, Lillie E, Zarin W, et al. PRISMA Extension for Scoping Reviews (PRISMA-ScR): Checklist and Explanation. Ann Intern Med. 2018 ;169(7):467-73. Doi: 10.7326/M18-0850.
11. Peters MDJ, Godfrey C, McInerney P, et al. Chapter 11: Scoping Reviews (2020 version). In: Aromataris E, Munn Z (Editors). Joanna Briggs Institute Reviewer's Manual; 2020. Available from https://reviewersmanual.joannabriggs.org/

For peer review only

12. Ferraz LT, Santos AJT, Lorenzi LJ, et al. Paper and screen media in current health education practices aimed at older adults: a scoping review protocol. BMJ Open 2023;13:e068762. doi:10.1136/ bmjopen-2022-068762
13. Haddaway NR, Collins AM, Coughlin D, Kirk S (2015) The Role of Google Scholar in Evidence Reviews and Its Applicability to Grey Literature Searching. PLoS ONE 10(9): e0138237. doi:10.1371/journal.pone.0138237
14. Bardin L. Content analysis. Lisboa: Edições, 2009.
15. Loveys, K., Sagar, M., Pickering, I., & Broadbent, E. (2021). A digital human for delivering a remote loneliness and stress intervention to at-risk younger and older adults during the COVID-19 pandemic: Randomized pilot trial. JMIR mental health, 8(11), e31586.
16. Chin, J., Moeller, D. D., Johnson, J., Duwe, E. A., Graumlich, J. F., Murray, M. D., & Morrow, D. G. (2018). A multi-faceted approach to promote comprehension of online health information among older adults. The Gerontologist, 58(4), 686-695.
17. Pot, A. M., Blom, M. M., & Willemse, B. M. (2015). Acceptability of a guided self-help Internet intervention for family caregivers: mastery over dementia. International psychogeriatrics, 27(8), 1343-1354.
18. Crandall, K. J., Shake, M., & Ziegler, U. (2019). Assessing the Impact of a Game-Centered Mobile App on Community-Dwelling Older Adults′ Health Activation. OBM integrative and complimentary medicine, 4(3).
19. Ogrin, R., Viswanathan, R., Aylen, T., Wallace, F., Scott, J., & Kumar, D. (2018). Co-design of an evidence-based health education diabetes foot app to prevent serious footcomplications: a feasibility study. Practical diabetes, 35(6), 203-209d.
20. Vanoh, D., Ishak, I. H., Shahar, S., Manaf, Z. A., Ali, N. M., & Noah, S. A. M. (2018). Development and assessment of a web-based intervention for educating older people on strategies promoting healthy cognition. Clinical interventions in aging, 1787-1798.
21. Im, C., & Park, M. (2014). Development and evaluation of a computerized multimedia approach to educate older adults about safe medication. Asian Nursing Research, 8(3), 193- 200.
22. Jongstra, S., Beishuizen, C., Andrieu, S., Barbera, M., van Dorp, M., van de Groep, B., ... & Richard, E. (2017). Development and validation of an interactive internet platform for older people: the healthy ageing through internet counselling in the elderly study. Telemedicine and e-Health, 23(2), 96-104.
23. Koops van't Jagt, R., de Winter, A. F., Reijneveld, S. A., Hoeks, J. C., & Jansen, C. J. (2016). Development of a communication intervention for older adults with limited

health literacy: Photo stories to support doctor–patient communication. Journal of Health Communication, 21(sup2), 69-82.

24. Peels, D. A., De Vries, H., Bolman, C., Golsteijn, R. H. J., Van Stralen, M. M., Mudde, A. N., & Lechner, L. (2013). Differences in the use and appreciation of a web-based or printed computer-tailored physical activity intervention for people aged over 50 years. Health Education Research, 28(4), 715-731.
25. Mouton, A., & Cloes, M. (2015). Efficacy of a web-based, center-based or combined physical activity intervention among older adults. Health Education Research, 30(3), 422- 435
26. LaMonica, H. M., Roberts, A. E., Davenport, T. A., & Hickie, I. B. (2021). Evaluation of the usability and acceptability of the InnoWell platform as rated by older adults: survey study. JMIR aging, 4(2), e25928.
27. Xie, B. (2012). Improving older adults'e-health literacy through computer training using NIH online resources. Library & information science research, 34(1), 63-71.
28. Chung, K., Kim, S., Lee, E., & Park, J. Y. (2020). Mobile app use for insomnia selfmanagement in urban community-dwelling older Korean adults: retrospective intervention study. JMIR mHealth and uHealth, 8(8), e17755.
29. Olson, E. A. (2014). Self-efficacy, self-regulation, and physical activity behavior in type 2 diabetes. University of Illinois at Urbana-Champaign.
30. Pandrangi, V. C., Gaston, B., Appelbaum, N. P., Albuquerque Jr, F. C., Levy, M. M., & Larson, R. A. (2019). The application of virtual reality in patient education. Annals of vascular surgery, 59, 184-189.
31. Jansen-Kosterink, S., Varenbrink, P., & Naafs, A. (2018). The GezelschApp a Dutch mobile application to reduce social isolation and loneliness. In 4th International Conference on Information and Communication Technologies for Ageing Well and e-Health, ICT4AWE 2018 (pp. 142-147). SCITEPRESS.
32. Jones, A. S., Fernandez, J., Grey, A., & Petrie, K. J. (2017). The impact of 3-D models versus animations on perceptions of osteoporosis and treatment motivation: a randomised trial. Annals of Behavioral Medicine, 51(6), 899-911.
33. Wei, C. W., Kao, H. Y., Wu, W. H., Chen, C. Y., & Fu, H. P. (2021). The influence of robotassisted learning system on health literacy and learning perception. International Journal of Environmental Research and Public Health, 18(21), 11053.
34. Marino, R. J., Marwaha, P., & Barrow, S. Y. (2016). Web-based oral health promotion program for older adults: Development and preliminary evaluation. International journal of medical informatics, 91, e9-e15.
35. Pischke, C. R., Voelcker-Rehage, C., Ratz, T., Peters, M., Buck, C., Meyer, J., ... & Lippke, S. (2022). Web-based versus print-based physical activity intervention for community-dwelling older adults: crossover randomized trial. JMIR mHealth and uHealth, 10(3), e32212.
36. World Health Organization. (2021a). Global strategy on digital health 2020-2025.

For peer review only

37. Morey, S. A., Stuck, R. E., Chong, A. W., Barg-Walkow, L. H., Mitzner, T. L., & Rogers, W. A. (2019). Mobile health apps: improving usability for older adult users. *Ergonomics in Design*, *27*(4), 4-13.

38. Leme, R. R. (2015). Uma proposta de design da interação multimodal para e com a terceira idade para dispositivos móveis.

39. De Paiva, D. C.; Alves, H. V.. Evolução Tecnológica e as Diferentes Gerações. 2018.[1]

40. Wilson, J., Heinsch, M., Betts, D., Booth, D., & Kay-Lambkin, F. (2021). Barriers and facilitators to the use of e-health by older adults: a scoping review. *BMC public health*, *21*, 1-12.

41. Alruwaili, M. M., Shaban, M., & Elsayed Ramadan, O. M. (2023). Digital health interventions for promoting healthy aging: a systematic review of adoption patterns, efficacy, and user experience. *Sustainability*, *15*(23), 16503.

42. Maskell, K., McDonald, P., & Paudyal, P. (2018). Effectiveness of health education materials in general practice waiting rooms: a cross-sectional study. *British Journal of General Practice*, *68*(677), e869-e876.

43. Frohlich, D. M. (2017, October). From Audio Paper to Next Generation Paper. In *Proceedings of the 23rd Brazillian Symposium on Multimedia and the Web* (pp. 9-10).

44. Frohlich D, Corrigan-Kavanagh E, Bober M, et al. The Cornwall a-book: An augmented travel guide using next generation paper. The Journal of Electronic Publishing 2019;22(1). DOI: https://doi.org/10.3998/3336451.0022.101.

45. Bairaktaris, G., Frohlich, D., & Sporea, R. (2021, May). Printed Light Tags and the Magic Bookmark: Using light to augment paper objects. In *Extended Abstracts of the 2021 CHI Conference on Human Factors in Computing Systems* (pp. 1-5).

46. Frohlich, D. M., Ferraz, L., & Castro, P. (2022). Printed digital storytelling for older audiences [2]

For peer review only

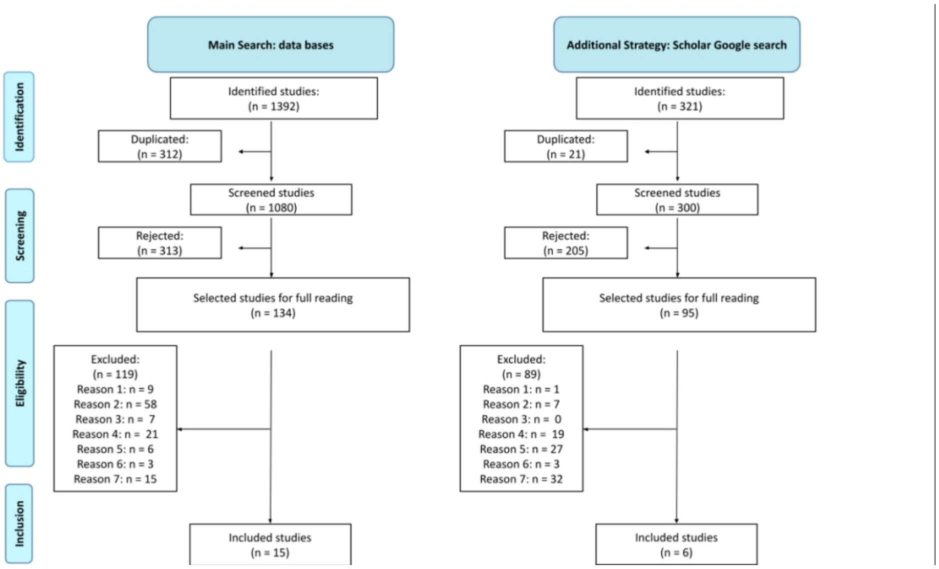

Description: Reason 1: it is a protocol, literature review or it has only an abstract available; Reason 2: it does not present the mean age or the mean age is less than 60 years; Reason 3: it does not have participants; Reason 4: it does not have a health education or the health education is not for promotion or prevention; Reason 5: health education does not use paper or screen-based media; Reason 6: it does not describe the health education; Reason 7: it does not present barriers or facilitators perceived by users in relation to the media used.
Source: developed by the authors.

234x131mm (144 x 144 DPI)

Preferred Reporting Items for Systematic reviews and Meta-Analyses extension for Scoping Reviews (PRISMA-ScR) Checklist

SECTION	ITEM	PRISMA-ScR CHECKLIST ITEM	REPORTED ON PAGE #
**TITLE**			
Title	1	Identify the report as a scoping review.	
**ABSTRACT**			
Structured summary	2	Provide a structured summary that includes (as applicable): background, objectives, eligibility criteria, sources of evidence, charting methods, results, and conclusions that relate to the review questions and objectives.	
**INTRODUCTION**			
Rationale	3	Describe the rationale for the review in the context of what is already known. Explain why the review questions/objectives lend themselves to a scoping review approach.	
Objectives	4	Provide an explicit statement of the questions and objectives being addressed with reference to their key elements (e.g., population or participants, concepts, and context) or other relevant key elements used to conceptualize the review questions and/or objectives.	
**METHODS**			
Protocol and registration	5	Indicate whether a review protocol exists; state if and where it can be accessed (e.g., a Web address); and if available, provide registration information, including the registration number.	
Eligibility criteria	6	Specify characteristics of the sources of evidence used as eligibility criteria (e.g., years considered, language, and publication status), and provide a rationale.	
Information sources*	7	Describe all information sources in the search (e.g., databases with dates of coverage and contact with authors to identify additional sources), as well as the date the most recent search was executed.	
Search	8	Present the full electronic search strategy for at least 1 database, including any limits used, such that it could be repeated.	
Selection of sources of evidence†	9	State the process for selecting sources of evidence (i.e., screening and eligibility) included in the scoping review.	
Data charting process‡	10	Describe the methods of charting data from the included sources of evidence (e.g., calibrated forms or forms that have been tested by the team before their use, and whether data charting was done independently or in duplicate) and any processes for obtaining and confirming data from investigators.	
Data items	11	List and define all variables for which data were sought and any assumptions and simplifications made.	
Critical appraisal of individual sources of evidence§	12	If done, provide a rationale for conducting a critical appraisal of included sources of evidence; describe the methods used and how this information was used in any data synthesis (if appropriate).	
Synthesis of results	13	Describe the methods of handling and summarizing the data that were charted.	

For peer review only

SECTION	ITEM	PRISMA-ScR CHECKLIST ITEM	REPORTED ON PAGE #
**RESULTS**			
Selection of sources of evidence	14	Give numbers of sources of evidence screened, assessed for eligibility, and included in the review, with reasons for exclusions at each stage, ideally using a flow diagram.	
Characteristics of sources of evidence	15	For each source of evidence, present characteristics for which data were charted and provide the citations.	
Critical appraisal within sources of evidence	16	If done, present data on critical appraisal of included sources of evidence (see item 12).	
Results of individual sources of evidence	17	For each included source of evidence, present the relevant data that were charted that relate to the review questions and objectives.	
Synthesis of results	18	Summarize and/or present the charting results as they relate to the review questions and objectives.	
**DISCUSSION**			
Summary of evidence	19	Summarize the main results (including an overview of concepts, themes, and types of evidence available), link to the review questions and objectives, and consider the relevance to key groups.	
Limitations	20	Discuss the limitations of the scoping review process.	
Conclusions	21	Provide a general interpretation of the results with respect to the review questions and objectives, as well as potential implications and/or next steps.	
**FUNDING**			
Funding	22	Describe sources of funding for the included sources of evidence, as well as sources of funding for the scoping review. Describe the role of the funders of the scoping review.	

JBI = Joanna Briggs Institute; PRISMA-ScR = Preferred Reporting Items for Systematic reviews and Meta-Analyses extension for Scoping Reviews.
* Where *sources of evidence* (see second footnote) are compiled from, such as bibliographic databases, social media platforms, and Web sites.
† A more inclusive/heterogeneous term used to account for the different types of evidence or data sources (e.g., quantitative and/or qualitative research, expert opinion, and policy documents) that may be eligible in a scoping review as opposed to only studies. This is not to be confused with *information sources* (see first footnote).
‡ The frameworks by Arksey and O'Malley (6) and Levac and colleagues (7) and the JBI guidance (4, 5) refer to the process of data extraction in a scoping review as data charting.
§ The process of systematically examining research evidence to assess its validity, results, and relevance before using it to inform a decision. This term is used for items 12 and 19 instead of "risk of bias" (which is more applicable to systematic reviews of interventions) to include and acknowledge the various sources of evidence that may be used in a scoping review (e.g., quantitative and/or qualitative research, expert opinion, and policy document).

From: Tricco AC, Lillie E, Zarin W, O'Brien KK, Colquhoun H, Levac D, et al. PRISMA Extension for Scoping Reviews (PRISMAScR): Checklist and Explanation. Ann Intern Med. 2018;169:467–473. doi: 10.7326/M18-0850.

St. Michael's
Inspired Care. Inspiring Science.

For peer review only - http://bmjopen.bmj.com/site/about/guidelines.xhtml

Design considerations for the migration from paper to screen media in current health education for older adults: A scoping review

Journal:	*BMJ Open*
Manuscript ID	bmjopen-2023-078647.R2
Article Type:	Original research
Date Submitted by the Author:	23-Feb-2024
Complete List of Authors:	Ferraz, Larissa; University of Surrey, Music and Media<br>Santos, Anna Julia ; Federal University of São Carlos, Department of Gerontology<br>Lorenzi, Lorena; Universidade Federal de São Carlos, Gerontology<br>Frohlich, David ; University of Surrey, Department of Music and Media<br>Barley, Elizabeth; University of Surrey, Mental Health Sciences and Nursing<br>Castro, Paula; UFSCar, Gerontology
**Primary Subject Heading**:	Public health
Secondary Subject Heading:	Health policy, Health services research
Keywords:	Health Education, Aged, BIOTECHNOLOGY & BIOINFORMATICS, Health informatics < BIOTECHNOLOGY & BIOINFORMATICS, PUBLIC HEALTH, Primary Prevention

SCHOLARONE™
Manuscripts

BMJ

I, the Submitting Author has the right to grant and does grant on behalf of all authors of the Work (as defined in the below author licence), an exclusive licence and/or a non-exclusive licence for contributions from authors who are: i) UK Crown employees; ii) where BMJ has agreed a CC-BY licence shall apply, and/or iii) in accordance with the terms applicable for US Federal Government officers or employees acting as part of their official duties; on a worldwide, perpetual, irrevocable, royalty-free basis to BMJ Publishing Group Ltd ("BMJ") its licensees and where the relevant Journal is co-owned by BMJ to the co-owners of the Journal, to publish the Work in this journal and any other BMJ products and to exploit all rights, as set out in our licence.

The Submitting Author accepts and understands that any supply made under these terms is made by BMJ to the Submitting Author unless you are acting as an employee on behalf of your employer or a postgraduate student of an affiliated institution which is paying any applicable article publishing charge ("APC") for Open Access articles. Where the Submitting Author wishes to make the Work available on an Open Access basis (and intends to pay the relevant APC), the terms of reuse of such Open Access shall be governed by a Creative Commons licence – details of these licences and which Creative Commons licence will apply to this Work are set out in our licence referred to above.

Other than as permitted in any relevant BMJ Author's Self Archiving Policies, I confirm this Work has not been accepted for publication elsewhere, is not being considered for publication elsewhere and does not duplicate material already published. I confirm all authors consent to publication of this Work and authorise the granting of this licence.

For peer review only

TITLE PAGE

Design considerations for the migration from paper to screen media in current health education for older adults: A scoping review

Larissa Taveira Ferraz[1]

Anna Julia Tavares Santos[2]

Lorena Jorge Lorenzi[3]

David M. Frohlich[1]

Elizabeth A. Barley[4]

Paula Costa Castro[2]

1. Department of Music and Media, University of Surrey, Guildford, United Kingdom.
2. Department of Gerontology, Federal University of São Carlos, São Carlos, Brazil.
3. Programa de Pós-graduação Interunidades em Bioengenharia, University of São Paulo, São Carlos, Brazil.
4. School of Health Sciences, University of Surrey, Guildford, United Kingdom

Corresponding author:
Larissa Taveira Ferraz

l.taveiraferraz@surrey.ac.uk

Digital World Research Centre, Room 15AP01, University of Surrey, Guildford GU2 7XH, UK

Keywords: Health education, Older people, Paper-based media, Screen-based media, Review.

Word count: 7218

For peer review only

Design considerations for the migration from paper to screen media in current health education for older adults: A scoping review

ABSTRACT

Objectives: To map the current use of paper-based and/or screen-based media for health education aimed at older people. **Design:** A scoping review was reported following the PRISMA-ScR checklist. **Data Sources:** The search was carried out in seven databases (Scopus, Web of Science, Embase, Cinahl, The ACM Guide to Computing Literature, Psyinfo), with studies available from 2012 to the date of the search in 2022, in English, Portuguese, Italian or Spanish, in addition to a Google Scholar search to check the grey literature. The terms used in the search strategy were older adults, health education, paper and screen-based media, preferences, intervention and other related terms. **Eligibility Criteria:** Studies were included that carried out health education interventions for older individuals using paper and/or screen-based media and that described barriers and/or facilitators of the use of these media. **Data extraction and synthesis:** The selection of studies was carried out by two reviewers. A data extraction form was developed with the aim of extracting and recording the main information from the studies. Data were analysed descriptively and using Bardin's content analysis. **Results:** The review included 21 studies that carried out health education interventions with different purposes, the main ones being promotion of physical activity, hypertension prevention and psychological health. All 21 interventions involved screen-based media on computers, tablets, smartphones and laptops, while only 4 involved paper-based media such as booklets, brochures, diaries, flyers and drawings. This appears to reflect a transition from paper to screen media for health education for an older population, in research if not practice. However, analysis of facilitators and barriers to use of both media revealed 10 design factors that could improve or reduce use, and complementarity in their application to each media type. For example, screen media could have multimedia Content, additional Functionality and interactivity through good Interaction Design, but have low Accessiblity and require additional Learning due to complex Interface Design. Conversely, paper media had static Content and low Functionality but high Accessibility, Availability and a low Learning overhead. **Conclusions**: We recommend improved screen media design, continued use of paper, and the possible combination of both media through the new augmented paper technology.

Registration number: Open Science Framework (DOI: DOI 10.17605/OSF.IO/GKEAH)

For peer review only

Strengths and limitations of this study

- It is the first review that mapped the differences in the use of paper-based and screen-based media for health education for older people.
- The studies were searched in seven databases, in addition to a grey literature search.
- The studies were verified in four languages, being Portuguese, English, Spanish and Italian.
- A limitation of the review was to have as an inclusion criterion that the studies should have a mean age equal to 60 years or older.

INTRODUCTION

The population of older adults aged 60 and over reached about 14% of the world's population in the year 2020, which means 1 in 7 people. In the year 1950, this percentage was 8% of the population.[1] Population ageing has been triggered by a series of factors, such as a decrease in the fertility rate and an increase in life expectancy.[2] The projection for 2050 is that adults over 60 will reach 22% of the world's population, that is, approximately 1 in 5 people.[1] This increase is also leading to more people living with chronic health conditions and a need to improve quality of later life through what is called healthy ageing.

The document entitled "Decade of Healthy Ageing", by the World Health Organization (WHO), proposes a plan to improve the health of the older adult population between 2021 and 2030.[3] It emphasises the importance of health conditions being managed efficiently with health promotion and disease prevention practices.[3] A key part of this strategy is the provision of effective health communication, through media that are disseminated both on paper and screen. According to WHO, health communication can be defined as "the use of communication strategies (eg, interpersonal, digital and other media) to inform and influence decisions and actions to improve health". [4]

Paper-based media are established formats for health education provided by professionals due to their widespread accessibility.[5] Such media were, and still are, widely used in health practices in the form of booklets, leaflets, posters and manuals.[6] In the literature, there are some studies that present recommendations and guidelines for their use, such as the one carried out by Hoffmann and Worrall (2004) [5], they emphasise the importance of these materials being: objective and updated; easy to understand for everyone; using table of contents, subheadings, bulleted lists, and abstracts; organised, with differentiation between

For peer review only

uppercase and lowercase letters and use of bold only for emphasis; with the use of images or drawings only to aid understanding; and with the use of interaction resources, for example a short questionnaire.

Increasingly, health education materials are being disseminated through applications and websites on screen-based devices such as smartphones and computers.[7] Many of these health education services are targeted at older adults, because they are disproportionately affected by illness and are likely to benefit most from information about healthy ageing practices. It should also be noted that, along with technological advances, there are other situations that enhance the use of digital media, such as the advent of the COVID-19 pandemic and the necessary social distancing.[8] Recommendations on the use and design of screen-based health information are also available in the literature. For example, the study by Zhao, Zhao and Song (2022) [9] explores the information seeking behaviour of older adults for health information and reveals that in addition to personal barriers, there are barriers in technology, such as unsatisfactory interactivity and usability, inadequate font sizes, dense texts, lack of visual elements and confusing layouts.

Current studies, including reviews, of the use of paper or screen formats for health information are carried out separately and seldom compared with each other. Furthermore, they address a wide range of ages in the audiences involved. The aim of this review is to map the use of paper and screen based media in current health education practices targeted at older people aged 60 or over. This should allow the identification of the barriers and facilitators in the use of each media for this population, comparing the use of these two types of media for health education.

METHODS AND ANALYSIS

This review was developed according to the guidelines of the *Preferred Reporting Items of Systematic Reviews and Meta-analyses for Scoping Reviews* (PRISMA-ScR) [10] and the recommendations of the Joanna Briggs Institute Revisions Manual (JBI). [11] The protocol of the review with methodological details is published.[12] The research questions were:

1. What types of paper-based and screen-based media are used in current forms of health education and how are they used for health education of older adults?
2. What are the barriers and facilitators to using paper-based and screen-based media for health information of older adults?

For peer review only

3. What are the differences between the use of paper-based and screen-based media for health education?

Eligibility, inclusion and exclusion criteria

Scientific articles that had as their objective or central theme a health education intervention carried out with paper and/or screen-based media for health promotion or prevention of older individuals were considered eligible. Studies were included that carried out or described health education interventions for health promotion or prevention of older individuals using paper and/or screen-based media and that described barriers and/or facilitators of the use of these media. The studies included should have a sample of subjects with a mean age of 60 years or older and be published in English, Portuguese, Spanish or Italian, from 2012 until the search date. In addition, grey literature studies were included through an additional search in the Google Scholar.

Studies that were not about health promotion or prevention, such as interventions on treating or caring for pre-existing health conditions were not included. In addition, studies that did not present barriers or facilitators perceived by users in relation to the media were excluded. Political documents, technical reports and studies whose full texts could not be obtained, even after attempts to contact the authors, were excluded.

Search Strategy

A search strategy was developed based on preliminary searches in databases, using the terms *older adults, health education, paper-based media, screen-based media, experience, intervention*, in addition to other related terms. The search strategy was adapted using Boolean operators and Mesh terms according to each database. The search strategy used in Scopus databases was:

("older person" OR elder* OR "older adults" OR "elderly population" OR "older people" OR ageing OR aging OR "older population" OR geriatric OR "healthy ageing" OR "successful aging") AND ("paper based media" OR "screen based media" OR website* OR platform* OR virtual OR online OR multimodal OR multimedia OR "reading media" OR "digital-based reading" OR "paper based reading" OR flyer OR "media advertisement" OR "print media" OR app OR apps OR tablet* OR smartphone* OR m-health* OR e-health* OR "patient information leaflets") AND ("health education" OR "health information" OR "health communication" OR "health promotion") AND (preference* OR characteristic* OR experience* OR attribute*

For peer review only - http://bmjopen.bmj.com/site/about/guidelines.xhtml

OR perception* OR development OR barrier* OR facilitator* OR opportunities OR problem* OR recommendations) AND (trial OR intervention)

Searches were performed in seven databases: Scopus (Elsevier), Web of Science (Clarivate), Medline, Embase (Elsevier), Cinahl (EBSCO), The ACM Guide to Computing Literature, Psyinfo (APA) databases. All search strategies used in the databases are described in the Supplementary File. Studies published in Portuguese, English, Spanish and Italian, from 2012 to the date of the search in June 2022, were searched. An additional search on Google Scholar was performed to verify 150 studies according to Google's relevance algorithm in the year 2022. Later, in April 2023, after guidance from the protocol reviewer that suggested the search should include the first 300 studies, based on the paper by Haddaway NR (2015),[13] the expansion of the grey literature was carried out, updating the search for April 2023, until the first 300 studies from Google Scholar were identified.

Study/Source of evidence selection

The selection of studies was carried out in five stages, starting with the search in the databases in which the identified studies were extracted into the Excel program and duplicates were removed. The next step was a pilot test in which two reviewers checked 25 random studies found in the search according to the eligibility criteria, obtaining a 92% agreement rate.

The third step was the reading of abstracts, titles and keywords of all studies by two reviewers independently following the eligibility criteria. And the fourth step was the reading of the full texts of the studies accepted in the previous step by the reviewers independently according to the inclusion criteria. In both phases, a third reviewer resolved disagreements. The last step was the additional strategy, which was the reading of the first 300 studies found in Google Scholar according to the relevance algorithm.

Data extraction

Data from the studies included in the review were extracted into a table developed and checked by the authors in three studies. The extracted data contained information about the studies such as authors' names, title, year of publication, country of origin and methods used, information about the sample such as size, gender and mean age, characteristics of the intervention such as duration and frequency, health condition verified, paper media used, screen media used, barriers to the use of paper and/or screen media and facilitators in the use of paper and/or screen media. The extraction table was described in the protocol for this scoping review, however the topic "Is the health education aimed at the caregiver?' was withdrawn.

For peer review only

This topic was removed from the extraction table, considering that throughout the study it was identified that the papers included were mostly focused on the participants 60+ and not caregivers.[13]

Data analysis and presentation

The data extracted from the included studies were presented in a narrative form and through tables and the PRISMA flowchart. Quantitative data were analysed using relative and absolute frequency, mean and standard deviation. Qualitative data about the barriers and facilitators in the use of paper and/or screen-based media were analysed using Bardin's content analysis.[14] Barriers and facilitators were grouped into categories according to their similarity.

RESULTS

The databases searched resulted in 1,392 studies, as shown in the flow diagram in figure 1. Duplicate studies were removed which resulted in 1,080 articles found. All the studies found were screened by the two reviewers, and according to the eligibility criteria, a total of 134 articles were sent to the reading step of the full texts. In the selection of studies by the inclusion criteria the total number of included studies was 15 and 119 were excluded as described in the flowchart (Figure 1). In the additional strategy, which was the reading of the 300 studies found in Google Scholar in order of relevance, it was identified that 21 were duplicates that had already been found in the search of the databases. Thus, in order to have 300 studies without duplicates, 321 studies were screened. As a result, 95 studies were selected for full reading and, subsequently, 6 were included based on the same inclusion criteria applied to full texts. Therefore, the main search included 15 studies and the Google Scholar search 6 studies, this review then included a total of 21 studies.

Figure 1: PRISMA flow diagram of the studies included in the review that presents screen-based and/or paper-based media for health education.

(FIGURE 1)

Description: Reason 1: it is a protocol, literature review or it has only an abstract available; Reason 2: it does not present the mean age or the mean age is less than 60 years; Reason 3: it does not have participants; Reason 4: it does not have a health education or the health education is not for promotion or prevention; Reason 5: health education does not use paper or screen-based media; Reason 6: it does not describe the health education; Reason 7: it does not present barriers or facilitators perceived by users in relation to the media used.

Source: developed by the authors.

For peer review only

Characterization of the studies

Descriptive characteristics of the included studies (n = 21), such as year of publication, country where it was carried out, year of data collection, methodology, intervention, sample size and average age of participants are described in Table 1.

Table 1 – Descriptive characteristics of the included studies

**Variables**	**n**	**%**
Year of publication		
2012	1	4.76
2013	1	4.76
2014	2	9.52
2015	2	9.52
2016	2	9.52
2017	2	9.52
2018	5	23.81
2019	2	9.52
2021	3	14.29
2022	1	4.76
Location - Country		
United States	5	23.81
Australia	3	14.29
Netherlands	3	14.29
New Zealand	2	9.52
South Korea	2	9.52
Belgium	1	4.76
Malaysia	1	4.76
Germany	1	4.76
Taiwan	1	4.76

For peer review only

International (several countries)	2	9.52
Year of data collection		
2007-2009	1	4.76
2010-2011	1	4.76
2012	2	9.52
2014-2015	1	4.76
2015	1	4.76
2016	1	4.76
2017	1	4.76
2017-2018	1	4.76
2020	1	4.76
2020-2021	1	4.76
NR	10	47.62
Methodology		
Longitudinal	17	80.95
Cross-sectional	4	19.05
Intervention		
In-person	11	52.38
Online	2	9.52
In-person and online	8	38.10
Sample size		
05-30	6	28.57
31-60	7	33.33
84	1	4.76
121-150	4	19.05
204	1	4.76
218	1	4.76

For peer review only

703	1	4.76
Average age of participants		
60-64 years	5	23.81
65-69 years	7	33.33
70-74 years	4	19.05
75-79 years	3	14.29
Unknown	2	9.52

NR: Not reported
Source: developed by the authors.

Regarding the year of publication described in table 1, approximately 61.9% of the articles were published in 2017, 2018, 2019, 2021 or 2022. Respectively, the United States, Australia, Netherlands, New Zealand and South Korea were the countries that most originated studies in this review. The year of data collection in the studies showed great variability, with the oldest year being 2007 and the most recent year being 2021. The majority of studies were carried out longitudinally (80.95%).

The interventions were mostly face-to-face (52.38%) and had a minimum duration of 30 minutes and a maximum duration of 6 months. The studies' sample ranged from 5 to 703 participants, however, mostly had 31 to 60 participants (33.33%) or 5 to 30 participants (28.57%) and most of the studies had an average age between 65 to 69 years old (33.33%) or 60 to 64 years old (23.81%). Two studies (9.52%) did not report mean age, but did not have adults (aged 18 to 59) in the sample. The mean total age of participants across all included studies was 68.51 (±5.06) years. Most studies (90.48%) were performed with individuals of both genders and the other studies did not report data on gender. In addition, 47.62% of the studies did not have adults (aged 18 to 59) in the sample, 47.62% of the studies had adults, and one study did not report this information, but the mean age was over 60 years.

Health education themes and types of media found in the studies

In response to the first research question of this review about the *formats* of health information used and for what purposes, Table 2 presents all the studies included in this scoping review, with their authors, year of publication, central theme of the health education and what type of digital/ paper media format was used.

For peer review only

The central theme of health education had great variability among the included studies, however it was identified that the themes that most appeared were physical activity promotion (14.29%), psychological health (14.29%) and hypertension prevention and care (9.52%).

Among the screen-based media formats used for health education in the studies, the following media were identified: website (61.90%), app (23.81%), email (9.52%) and animations on Microsoft PowerPoint (4.76%). These media were used on the devices: computers (52.38%), tablets (28.57%), smartphones (19.05%) and laptops (4.76%). Some studies used more than one media or device. Regarding the paper-based media formats used for health education in the studies, it was identified that they were booklets (A4 Paper), brochures, handouts, diaries, flyers, drawings and print-delivered information (without specification).

A major finding of the review was the imbalance of studies on paper and screen interventions. All 21 articles included in this study featured screen-based media, and among these, only 4 also reported the contrasting use of paper-based media. No studies were found that reported on the use of paper-based interventions alone.

Table 2: Studies included in the review, health education provided and types of paper-based and/or screen-based media used.

Article title	Authors, Year	Central theme of the health education	Screen-based Media Format	Paper-based Media format
A Digital Human for Delivering a Remote Loneliness and Stress Intervention to At-Risk Younger and Older Adults During the COVID-19 Pandemic: Randomized Pilot Trial [15]	Loveys et al., 2021	Mental health and well-being	Computer, tablet or smartphone (website)	NA (Not applicable)
A Multi-faceted Approach to Promote Comprehension of Online Health Information Among Older Adults[16]	Chin et al, 2018	Self-care regarding hypertension	Computer	NA
Acceptability of a guided self-help Internet intervention for family caregivers: Mastery over dementia [17]	Pot; Blom; Willemse, 2015	Psychological distress of family caregivers of people with dementia	(Website)	NA
Assessing the Impact of a Game-Centered Mobile App on Community-Dwelling Older Adults' Health Activation [18]	Crandall; Shake; Ziegler, 2019	Risk of falls and osteoarthritis	Tablet (App)	NA
Co-design of an evidence-based health education diabetes foot app to	Ogrin et al, 2018	Foot health for people with diabetes	Smartphone (app)	NA

For peer review only - http://bmjopen.bmj.com/site/about/guidelines.xhtml

prevent serious foot complications: a feasibility study[19]				
Development and assessment of a web-based intervention for educating older people on strategies promoting healthy cognition[20]	Vanoh et al, 2018	Precautionary strategies for mild cognitive impairment	Computer (Website)	NA
Development and Evaluation of a Computerized Multimedia Approach to Educate Older Adults about Safe Medication[21]	Im & Park, 2014	Safe medication	Computer (Website)	NA
Development and Validation of an Interactive Internet Platform for Older People: The Healthy Ageing Through Internet Counselling in the Elderly Study[22]	Jongstra et al, 2017	Improving the cardiovascular risk profile of old people with high cardiovascular risk	Computer (Website)	NA
Development of a communication intervention for older adults with limited health literacy: Photo stories to support doctor–patient communication[23]	Koops et al., 2016	Doctor-patient communication and communicative health literacy	(Website)	Booklet (A4 Paper)
Differences in the use and appreciation of a web-based or printed computer-tailored physical activity intervention for people aged over 50 years[24]	Peels et al, 2013	Physical activity promotion	Computer (Website; email)	Print-delivered information, brochures and handouts
Efficacy of a web-based, center-based or combined physical activity intervention among older adults[25]	Mouton & Cloes, 2015	Physical activity	(Website; email)	NA
Evaluation of the usability and acceptability of the InnoWell platform as rated by older adults: Survey study [26]	Lamonica et al 2021	Biopsychosocial domains	Tablet, computer, laptop, smartphone (Website)	NA
Improving older adults'e-health literacy through computer training using NIH online resources[27]	Xie; Bo, 2012	Improve the ability of seniors to search, find and understand health information online	Computer (website)	NA
Mobile App Use for Insomnia Self-Management in Urban Community-Dwelling Older Korean Adults: Retrospective Intervention Study[28]	Chung et al, 2018	Sleep education	Smartphone (app)	NA
Self-efficacy, self-regulation, and physical activity behavior in type 2 diabetes[29]	Olson, 2014	Metabolic Health Education	(Website)	NA

For peer review only

The Application of Virtual Reality in Patient Education[30]	Pandrangi et al, 2019	Health education - health status	(Website)	Flyers, drawings and brochures
The GezelschApp A Dutch Mobile Application to Reduce Social Isolation and Loneliness[31]	Jansen-Kosterink, Varenbrink & Naafs, 2018	Social isolation and loneliness among old people	Computer and tablet (App)	NA
The Impact of 3-D Models versus Animations on Perceptions of Osteoporosis and Treatment Motivation: A Randomised Trial[32]	Jones et al, 2017	Information about osteoporosis	Tablet (animations on Microsoft PowerPoint)	NA
The influence of robot-assisted learning system on health literacy and learning perception[33]	Wei et al, 2021	Hypertension prevention for older adults	Tablet and computer	NA
Web-based oral health promotion program for older adults: Development and preliminary evaluation[34]	Marino et al, 2016	Oral health promotion	Computer (Website)	NA
Web-based versus print-based physical activity intervention for community-dwelling older adults: crossover randomized trial[35]	Pischke et al., 2022	Promoting a physically active (PA) lifestyle	(Website and app)	Brochures and diary

NA: Not applicable.
Source: developed by the authors.

Categorization of barriers and facilitators

In response to the second research question about the barriers and facilitators to use, it was identified that the vast majority were about screen-based media. Barriers were considered to be difficulties that older people had when using screen or paper media, factors that hindered the use or the lack of features. Facilitators, on the other hand, were factors that contributed or motivated individuals to use the media. Thus, they were grouped according to their similarity into 10 categories, described below:

- Accessibility - the diversity of users able to access the media system.
- Availability - the availability to use the media system.
- Content design - the design of the media content itself.
- Functionality - what the media system does.
- Interaction design - design of the sequential organisation of the interaction with the media system.
- Interface design - design of the input and output interfaces.

For peer review only

- Learning - the ease of learning to use the media system.
- Privacy - privacy of personal data in the media system.
- Social interaction - the support of interaction with other people around the media system.
- Usability - the ease of using the media system.

The topics defined for the categorisation of barriers and facilitators seem to be properties of the design of media systems in general that can have both negative (barriers) and positive (facilitators) effects. They are different sides to the same coin because if done well they improve health education, but if done badly they reduce it. The categories found in barriers and facilitators related to screen-based and paper-based media are described in Table 3. The table describes the number of studies that identified each category, with some studies finding more than one type of category as barriers and/or facilitators. In the following sub-sections we step through the cited barriers and facilitators for each medium in turn.

Table 3 - Barriers and facilitators found in the included studies.

Topics	Screen-Based Media		Paper-Based Media	
	Barriers	Facilitators	Barriers	Facilitators
**Accessibility**	5	3	--	--
**Availability**	1	3	--	2
**Content design**	4	8	--	1
**Functionality**	3	6	2	--
**Interaction design**	2	4	--	--
**Interface design**	2	4	--	--
**Learning**	6	4	--	--
**Privacy**	1	--	--	--
**Social interaction**	1	2	--	--
**Usability**	3	6	--	--

Source: developed by the authors.

Barriers of screen-based media

The main categories that appeared as barriers to the use of screen-based media were Learning (e.g. lack of computer training, lack of instructional videos, the participants wanted

For peer review only - http://bmjopen.bmj.com/site/about/guidelines.xhtml

clear guidelines, older adults required technology training to be completed in-person), Accessibility (e.g. would prefer a larger screen using a tablet or laptop, prefer hard copies of the information, “a sad thing is you can’t touch her or talking to a real person”), Content design (e.g. strange contents, technical terms, information wasn’t as up to date, participants wanted varied levels of information), Functionality (e.g. lack of reward, some technical improvements are needed like the function to delete messages, defining a goal was difficult for the participants, an icon to facilitate printing would be useful) and Usability (e.g. the assignments were not direct and required big effort, passwords provided to login for the first time were too difficult). However, the following barriers were also identified: Availability (e.g. computer availability and computer reserves), Interaction design (e.g. aspects participants felt could be improved were the human likeness of it interaction like behaviours and voice and aspects of the conversation design), Interface design (e.g. videos were not easy to see because they had small size, small font size, lack of attractive photos and project colours), Privacy (e.g. participants did not post on the discussion board due to privacy concerns), Social interaction (e.g. limited app interaction with participants).

Facilitators of screen-based media

The main categories that appeared as facilitators for the use of screen-based media were Content design (e.g. information with revised content by researchers and experts helped them to understand the content, easy-to-understand text structures, bulleted lists, and phrases, animation based on a life story of an older adult character contributed to enhancing the self-efficacy of the participants), Usability (e.g. operating the program just by clicking the mouse was easy, it was simple and easy to navigate on a mobile phone, easy to use interface, most participants found these sites easy or very easy to use), Functionality (e.g. participants really liked the function to be able to make new friends and send and receive messages, participants mentioned videos as the most useful components, feedback was helpful), Accessibility (e.g. the app was appropriate for people with a low level of education, touch screen facilitates the use by old adults) and Interaction design (e.g. participants appreciated the interactive features of the platform like the interactive videos, interactive questions help with problem-solving, participants liked the interpersonal skills). However, the following facilitators were also identified: Availability (e.g. information on the smartphone can be seen at any time quickly, permanent access to the website), Interface design (e.g., the animations on the tablet allowed more of the information to be visualised, participants liked the appearance, cross-referenced a number of the images and statements to various sections), Learning (e.g. participants reported

For peer review only

that they easily learned how to use the application,, they also enjoyed and felt comfortable with self-learning), Social interaction (e.g. many participants expressed how much they enjoyed the social aspect of the app, participants considered the app a pleasant and safe way to find new friends).

Barriers of the use of paper-based media

Regarding the barriers related to paper-based media, the study by Pischke et al (2022) [35] found Functionality to be a barrier as the printed version seemed to be impractical regarding physical activity. Pandrangi et al (2019) [30] also identified Functionality as a barrier since participants perceived paper as a traditional method and considered virtual reality more useful.

Facilitators of the use of paper-based media

In the study by Koops et al. (2016) [23] it was identified that the paper was a facilitator in relation to Availability, as the participants reported that they could take it anywhere and Content design, as they liked that the stories were similar to real life and that it was easier for them to understand. Peels et al. (2013) [24] found availability to be a facilitator since the participants had more intentions to keep the information and to use the concepts learned in the future.

Differences between the use of paper-based and screen-based media for health education

Regarding the third research question about the differences between the use of paper-based and screen-based media, this was difficult to answer because of the small number of direct comparisons made. However, three particular barrier/facilitator categories appeared to relate to the contrasting properties of paper and screen, as expressed in the four studies using paper and screen materials. These were Availability, Functionality and Learning.

Availability appeared in two studies as facilitators concerning the use of paper media due to the possibility of having the paper in hand and being able to use it at any time. For the use of screen-based media, there is a need for more effort, for example, when having to turn on

For peer review only

a computer, log in to a website, download an application, etc., in order to be able to start using this media. [23, 24]

Considering the use of paper-based media, two other studies identified Functionality as a barrier to their use, due to the fact that this is limited compared to screen media, in which there is the possibility of having different functions, such as having feedback, interactive videos, setting goals and being able to make new friends. [30, 35]

In addition, the Learning category is related to the difference in the use of paper and screen due to the older audience having individuals who have difficulty using screen media, as they have no affinity with it and need training to use them. [35, 25] Thus, for individuals with difficulty in relation to the use of screen media, the use of paper is preferable, as seen in the study by Pischke et al. (2022). [35]

DISCUSSION

The review identified 21 studies that carried out health education interventions to promote ways of staying healthy longer to an ageing population subject to more chronic health conditions, the main subjects in the interventions were related to promotion of physical activity, hypertension prevention and psychological health. This was reflected in the health education subjects of the papers . These were directed to issues such as metabolic health, sleep, healthy eating, physical activity and cognition, and focused on the development and maintenance of autonomy and independence, enabling healthy ageing, according to the WHO definition. [3]

The interventions were done through the use of screen-based media such as websites, apps and emails, on desktop computers, tablets, smartphones and laptops, and paper-based media such as booklets, brochures, diaries, flyers and drawings. We found that the barriers and facilitators to the use of these media were essentially design properties that could improve media use if done well or reduce use if done badly. This led us to propose a taxonomy of 10 design features and considerations which apply to both media, somewhat unequally. The main design considerations for the use of screen-based media were related to Content design, Functionality, Usability, Accessibility and Learning. The main design considerations for the use of paper-based media, were Availability and Content design, but such media were often considered traditional because they did not have extra functionality.

We can see from the diversity of paper and screen materials in the studies that these terms each refer to quite different formats. In fact, they gloss these differences and do not really do justice to the different interactions people could have with each format. This may be one

For peer review only

reason for the ambiguity of findings in such comparisons in the past, and suggests greater attention to *particular* paper and screen formats in future research. For example, screen information was presented on four physical devices in the above studies, including desktop and laptop computer, tablet and smartphone. Sometimes this information was on a website or presented in a more interactive app, leading to a matrix of 8 possibilities across devices with different user experiences. Likewise, paper information was presented on single page leaflets, fliers and handouts, as well as multi-page booklets or diaries. Each of these has different affordances for reading, finding, handling and capturing information, and are not equivalent user experiences. Further work is required to understand the subtleties of design, interaction and experience will all these ‘paper’ and ‘screen’ formats, since the current papers do not go into sufficient detail.

Regarding the types of digital media used for health education, the World Health Organization has a 'Global Strategy on Digital Health 2020-2025' [36] with the purpose of strengthening health systems through digital health technology and this will engage patients to achieve the vision of health and well-being for all. This strategy signals that practices for public awareness of digital health must be addressed, this includes health literacy and patient engagement. [36] Our findings clearly reveal a noticeable trend towards digitization and a preference for screen-based media in the literature. When analysing the review data from the last decade, we observed a significant increase in the number of studies and publications focusing on screen-based media across all years, as depicted both in Tables 1 and 2. Our study found barriers and facilitators that, if properly addressed, will make new digital health strategies more user friendly for older people, which will improve the engagement of this group in health education interventions and consequently improve health literacy. Key amongst these were the design of *functionality* and *content*, which were shown to have a great impact, both positive and negative, on interventions.

Based on the findings of our review, the discussion in the literature emphasises the significance of interface design and screen media usability. Morey et al. (2019) [37] identified issues with an excess of icons and low contrast, highlighting participant concerns. Vanoh et al.'s (2018)[20] study underscored that older users prefer visually appealing media featuring attractive photos and colours, while Jongstra et al. (2017)[22] revealed their need for different functionalities. Hence, it is imperative to incorporate diverse functionalities tailored to the requirements of the older population, coupled with an engaging design characterised by colour contrast and intuitiveness to enhance usability.

For peer review only

The adoption of a user-centred design approach is crucial to meet the unique needs of older audiences. Co-design sessions, as suggested by Leme (2014)[38], serve as an effective strategy for involving older individuals in the creation and design processes of new tools across various media. In this way, participatory design enables the creation of intuitive media, which, consequently, older individuals will find easy to use. Moreover, it was identified in the included studies that one of the main barriers was the difficulty of learning these screen media. Therefore, it is crucial that public policies are developed and improved for the digital inclusion of older people, such as programs that teach these individuals how to use these media, so that they can enjoy all the benefits of digital technologies. The study by Chung et al. (2018)[28], which aims to analyse the usability of a sleep education app for older Koreans, presents as a perceived facilitator by users the ease with which they learned how to use the application.

Regarding screen media use, learning to use the media was one of the most prevalent barriers. According to De Paiva and Alves (2018) [39], the current population of older users accompanied the emergence of the internet, the printer and the computer. Nowadays, with a technological generation, games with different levels of proficiency and the emergence of artificial intelligence, the older population finds it difficult to adapt.

In one of the studies of this review, by Pischke et al. (2022)[35], that compared the acceptance and effectiveness of two interventions for physical activity (PA) promotion for older adults through websites, apps, brochures and diaries, they found individuals who have no affinity with screen media, preferring paper. In another study found in this review, by Peels et al. (2013)[24], based on a perception considered an Usability barrier, that reports "The assignments integration into the web-based tailored advice were not direct and required big effort to participate", it is notable that not being intuitive leads to a greater amount of effort. Thus, older people have greater difficulty with screen-based media and require more training. This training requirement is urgent at the current moment, given that the current young population, who are the future older people, are already growing up with skills and will likely have an easier time using them.[24]

Additionally, functionality is identified as a barrier in the use of e-health for older adults, as noted in Wilson's (2021)[40] scoping review. Conversely, ease of use emerged as a facilitator, as observed in both Alruwaili's (2023)[41] and Wilson's (2021)[40] studies, aligning with our usability review category.

Our study's results align with concerns mentioned in Olson's (2014)[29] research, particularly regarding privacy, which is also identified as a barrier in related studies such as Alruwaili's, 2023[41] systematic review and Wilson's, 2021[40] study. Privacy and data

For peer review only

security apprehensions are recognised as obstacles to digital health interventions among older adults. Therefore, it is essential to create public policies that ensure the privacy and security of users' data when using screen-based media in health education interventions. It is also important that policies regarding screen media are presented to the user at the beginning of the interaction with the media, as this will inform and ensure safety for users.

Digital engagement carries the danger and opportunity of data capture and user profiling. The Global Strategy on Digital Health 2020-2025[36] emphasises that health data is sensitive and personal, it requires a high standard of security and protection. Lack of trust and confidence about personal health data can discourage people from fully participating in health education interventions. WHO mentions the need for a strong legal and regulatory foundation to protect privacy, integrity, confidentiality and deal with data cyber security. It is also important to maintain transparency and communicate effectively about data security strategies to users. [36]

Regarding the use of paper media for health education, according to the findings of this review, it is clear that these media are still very much alive today, as revealed in the study by Koops et al. (2016) [23], that used a website and a booklet to development a doctor-patient communication for older adults with limited health literacy. In this study, a booklet with photographic stories that aided in the communication between doctor and patient was considered to have good availability, as it could be given out and used anywhere. This is likely to reflect a pervasive practice of displaying posters and giving out leaflets in GP surgeries and hospitals across the globe, regardless of their lack of representation in this research review. Indeed, this is mentioned in the wider literature. This is supported by wider literature, such as a study by Maskell, McDonald and Paudyal (2018)[42], which found that more than three-quarters of patients noticed printed materials in the waiting rooms of general clinics in England, and a large proportion read them. Hence, paper-based media are already widely accepted and available as part of modern health education practices.

Regarding the international reach of these studies we note that they come largely from high income countries as shown in Table 1. There is a lack of research and representation from low and middle income countries, especially those with vulnerable populations, and large countries with vast and remote regions facing challenges in accessing healthcare services, as shown in Table 1. For populations with limited education and financial vulnerability, paper-based materials appear more accessible due to their traditional usability and cost-effectiveness. However, for individuals who are actually illiterate, screen readers and audio content, for instance, offer a level of accessibility that paper cannot match. These functionalities are

For peer review only

facilitators found in screen-based media seen in this review, as videos mentioned as the most useful components in the study of Olson (2014)[29]. Moreover, advancements in satellite connection technologies are now integrating previously isolated areas into the global network, potentially granting access to health information for a substantial number of people who currently lack access to up-to-date pamphlets and leaflets. This integration and interactivity brought about by screen-based media could also enable these individuals to connect with health teams and professionals in more densely populated areas. This, in turn, could lead to more personalised and humanised health education guidelines and interventions through technology. Overall, these developments hold promise in bridging the healthcare information gap and fostering improved health outcomes for underserved communities.

Given the advantages and disadvantages of paper and screen-based information emerging from this review, it may be valuable to combine them in future health campaigns, perhaps at a new level of granularity. This can be done through *augmented paper* technology which supports the reading of printed hotlinks on a nearby smartphone.[43] This results in a paper-and-screen reading experience in which users move back and forth between media which have been carefully designed to complement each other. For example, a printed travel guide can play interviews with local characters, video walks through the landscape, and trigger phone calls to organisations or show updated timetable information.[44] The user in this scenario does not have to know how to use the web to access the digital information. They simply take a picture of the printed page, speak its page number, turn the page or move a bookmark to access the links.[45] A similar approach could be taken to the design of augmented patient information leaflets, posters and cards, which could play multimedia illustrations, spoken translations or instructions, and call local clinics or medical services for more advice.[46]

This review is limited to studies reporting the use of participants with a mean age equal to 60 years or older. Thus, studies that had not reported the mean age or age range of participants were excluded, but it may be that some would have been included if they had reported this information. Another limitation we encountered was the scarcity of studies exclusively utilising paper-based media, as none were solely dedicated for this form, and only 4 out of 21 studies found contrasted paper with screen media. Following the completed review, we identified 'printed material' as a potential descriptor that other researchers can use. While this trend might indicate the increasing use of screen media, it limits the enhancement of comparisons between the barriers and facilitators of both. In addition, a methodological bias was the inclusion criteria that participants should have reported barriers or facilitators, which resulted in a reduction in the number of articles included. Furthermore, as it is a scoping review,

For peer review only

its objective is to map all studies on the topic, therefore, an assessment of the quality of the included studies was not carried out.

The strengths of this review are: this is the first study that identifies and compares digital and paper media for health education aimed at older people, as well as design considerations related to their use; studies were searched for in seven databases and in the grey literature; studies were searched for in four languages, namely Portuguese, English, Spanish and Italian; systematic methods were used for the selection of studies e.g. all titles, abstracts and full texts were screened by two reviewers independently.

CONCLUSION

A transition or migration appears to be taking place in the provision of health information from paper to screen. In this review we have examined recent literature on the design and use of such information for older people across these formats and found an over-representation of research on screen interventions. Although this suggests that the future lies in greater digital engagement, a careful reading of 21 papers shows many barriers to the use of screen-based information and corresponding facilitators to the use of paper information for health, especially for an older population and broader international context. We therefore recommend greater attention to the barriers of screen-based information across different devices and services, continued use of paper, and the possible combination of both media through new augmented paper technology.

ETHICS

Ethical approval is not applicable to this scoping review, it only gathered information from published literature.

Author Contributions: All authors have made substantial contributions to the development of the manuscript. LTF and AJTS conceptualised the research question and prepared the first draft of the manuscript. LTF, AJTS, LJL, PCC, DMF, EAB contributed to the refining of the study design and guided the review development. Also, LTF, AJTS, and LJL analysed the results and PCC, DMF, EAB contributed to the discussion. Also LTF, AJTS, LJL, PCC, DMF, EAB contributed to the writing, and revising this review. DMF assisted in the revision of the English.

For peer review only

Competing interests: The authors declare no conflict of interest.

Funding: LTF received funding from the University of Surrey Doctoral College (Breaking Barriers Studentship Award), AJTS received funding from São Paulo Research Foundation (FAPESP), N° 2022/08918-6 to the development of this review and LJL received funding from Coordination for the Improvement of Higher Education Personnel (CAPES) - Financial Code 001.

Data sharing statement: Data from this review are available in this manuscript and in the previously published protocol. As it is a scoping review, the sharing of other data is not applicable.

Patient and Public Involvement statement: None.

Acknowledgements: We would like to acknowledge University of Surrey Doctoral College, São Paulo Research Foundation (FAPESP), Coordination for the Improvement of Higher Education Personnel (CAPES) for funding.

For peer review only

REFERENCES:

1. United Nations, Department of Economic and Social Affairs, Population Division (2022). World Population Prospects: The 2022 Revision. 2022. Available from: https://population.un.org/dataportal/.
2. United Nations Department of Economic and Social Affairs, Population Division (2022). World Population Prospects 2022: Summary of Results. 2022b. UN DESA/POP/2022/TR/NO. 3.
3. World Health Organization. Decade of healthy ageing: baseline report. Geneva, 2020.
4. World Health Organization. Health promotion glossary of terms 2021. 2021
5. Hoffmann, T., & Worrall, L. (2004). Designing effective written health education materials: considerations for health professionals. Disability and rehabilitation, 26(19), 1166-1173.
6. Sá, G. G. D. M., Silva, F. L., Santos, A. M. R. D., Noleto, J. D. S., Gouveia, M. T. D. O., & Nogueira, L. T. (2019). Technologies that promote health education for the community elderly: integrative review. Revista latino-americana de enfermagem, 27.
7. Kampmeijer, R., Pavlova, M., Tambor, M., Golinowska, S., & Groot, W. (2016). The use of e-health and m-health tools in health promotion and primary prevention among older adults: a systematic literature review. BMC Health Services Research, 16(5), 467-479.
8. Celuppi IC, Lima GS, Rossi E, et al. An analysis of the development of digital health technologies to fight COVID-19 in Brazil and the world. Cad Saude Publica 2021;37(3):e00243220. Doi: 10.1590/0102-311X00243220.
9. Zhao YC, Zhao M, Song S. Online Health Information Seeking Behaviors Among Older Adults: Systematic Scoping Review. Journal of medical Internet research 2022; 24 (2): e34790. DOI: 10.2196/34790
10. Tricco AC, Lillie E, Zarin W, et al. PRISMA Extension for Scoping Reviews (PRISMA-ScR): Checklist and Explanation. Ann Intern Med. 2018 ;169(7):467-73. Doi: 10.7326/M18-0850.
11. Peters MDJ, Godfrey C, McInerney P, et al. Chapter 11: Scoping Reviews (2020 version). In: Aromataris E, Munn Z (Editors). Joanna Briggs Institute Reviewer's Manual; 2020. Available from https://reviewersmanual.joannabriggs.org/

For peer review only

12. Ferraz LT, Santos AJT, Lorenzi LJ, et al. Paper and screen media in current health education practices aimed at older adults: a scoping review protocol. BMJ Open 2023;13:e068762. doi:10.1136/ bmjopen-2022-068762
13. Haddaway NR, Collins AM, Coughlin D, Kirk S (2015) The Role of Google Scholar in Evidence Reviews and Its Applicability to Grey Literature Searching. PLoS ONE 10(9): e0138237. doi:10.1371/journal.pone.0138237
14. Bardin L. Content analysis. Lisboa: Edições, 2009.
15. Loveys, K., Sagar, M., Pickering, I., & Broadbent, E. (2021). A digital human for delivering a remote loneliness and stress intervention to at-risk younger and older adults during the COVID-19 pandemic: Randomized pilot trial. JMIR mental health, 8(11), e31586.
16. Chin, J., Moeller, D. D., Johnson, J., Duwe, E. A., Graumlich, J. F., Murray, M. D., & Morrow, D. G. (2018). A multi-faceted approach to promote comprehension of online health information among older adults. The Gerontologist, 58(4), 686-695.
17. Pot, A. M., Blom, M. M., & Willemse, B. M. (2015). Acceptability of a guided self-help Internet intervention for family caregivers: mastery over dementia. International psychogeriatrics, 27(8), 1343-1354.
18. Crandall, K. J., Shake, M., & Ziegler, U. (2019). Assessing the Impact of a Game-Centered Mobile App on Community-Dwelling Older Adults′ Health Activation. OBM integrative and complimentary medicine, 4(3).
19. Ogrin, R., Viswanathan, R., Aylen, T., Wallace, F., Scott, J., & Kumar, D. (2018). Co-design of an evidence-based health education diabetes foot app to prevent serious footcomplications: a feasibility study. Practical diabetes, 35(6), 203-209d.
20. Vanoh, D., Ishak, I. H., Shahar, S., Manaf, Z. A., Ali, N. M., & Noah, S. A. M. (2018). Development and assessment of a web-based intervention for educating older people on strategies promoting healthy cognition. Clinical interventions in aging, 1787-1798.
21. Im, C., & Park, M. (2014). Development and evaluation of a computerized multimedia approach to educate older adults about safe medication. Asian Nursing Research, 8(3), 193- 200.
22. Jongstra, S., Beishuizen, C., Andrieu, S., Barbera, M., van Dorp, M., van de Groep, B., ... & Richard, E. (2017). Development and validation of an interactive internet platform for older people: the healthy ageing through internet counselling in the elderly study. Telemedicine and e-Health, 23(2), 96-104.
23. Koops van't Jagt, R., de Winter, A. F., Reijneveld, S. A., Hoeks, J. C., & Jansen, C. J. (2016). Development of a communication intervention for older adults with limited

For peer review only

health literacy: Photo stories to support doctor–patient communication. Journal of Health Communication, 21(sup2), 69-82.

24. Peels, D. A., De Vries, H., Bolman, C., Golsteijn, R. H. J., Van Stralen, M. M., Mudde, A. N., & Lechner, L. (2013). Differences in the use and appreciation of a web-based or printed computer-tailored physical activity intervention for people aged over 50 years. Health Education Research, 28(4), 715-731.
25. Mouton, A., & Cloes, M. (2015). Efficacy of a web-based, center-based or combined physical activity intervention among older adults. Health Education Research, 30(3), 422- 435
26. LaMonica, H. M., Roberts, A. E., Davenport, T. A., & Hickie, I. B. (2021). Evaluation of the usability and acceptability of the InnoWell platform as rated by older adults: survey study. JMIR aging, 4(2), e25928.
27. Xie, B. (2012). Improving older adults'e-health literacy through computer training using NIH online resources. Library & information science research, 34(1), 63-71.
28. Chung, K., Kim, S., Lee, E., & Park, J. Y. (2020). Mobile app use for insomnia selfmanagement in urban community-dwelling older Korean adults: retrospective intervention study. JMIR mHealth and uHealth, 8(8), e17755.
29. Olson, E. A. (2014). Self-efficacy, self-regulation, and physical activity behavior in type 2 diabetes. University of Illinois at Urbana-Champaign.
30. Pandrangi, V. C., Gaston, B., Appelbaum, N. P., Albuquerque Jr, F. C., Levy, M. M., & Larson, R. A. (2019). The application of virtual reality in patient education. Annals of vascular surgery, 59, 184-189.
31. Jansen-Kosterink, S., Varenbrink, P., & Naafs, A. (2018). The GezelschApp a Dutch mobile application to reduce social isolation and loneliness. In 4th International Conference on Information and Communication Technologies for Ageing Well and e-Health, ICT4AWE 2018 (pp. 142-147). SCITEPRESS.
32. Jones, A. S., Fernandez, J., Grey, A., & Petrie, K. J. (2017). The impact of 3-D models versus animations on perceptions of osteoporosis and treatment motivation: a randomised trial. Annals of Behavioral Medicine, 51(6), 899-911.
33. Wei, C. W., Kao, H. Y., Wu, W. H., Chen, C. Y., & Fu, H. P. (2021). The influence of robotassisted learning system on health literacy and learning perception. International Journal of Environmental Research and Public Health, 18(21), 11053.
34. Marino, R. J., Marwaha, P., & Barrow, S. Y. (2016). Web-based oral health promotion program for older adults: Development and preliminary evaluation. International journal of medical informatics, 91, e9-e15.
35. Pischke, C. R., Voelcker-Rehage, C., Ratz, T., Peters, M., Buck, C., Meyer, J., ... & Lippke, S. (2022). Web-based versus print-based physical activity intervention for community-dwelling older adults: crossover randomized trial. JMIR mHealth and uHealth, 10(3), e32212.
36. World Health Organization. (2021a). Global strategy on digital health 2020-2025.

For peer review only

37. Morey, S. A., Stuck, R. E., Chong, A. W., Barg-Walkow, L. H., Mitzner, T. L., & Rogers, W. A. (2019). Mobile health apps: improving usability for older adult users. *Ergonomics in Design*, *27*(4), 4-13.

38. Leme, R. R. (2015). Uma proposta de design da interação multimodal para e com a terceira idade para dispositivos móveis.

39. De Paiva, D. C.; Alves, H. V.. Evolução Tecnológica e as Diferentes Gerações. 2018.[1]

40. Wilson, J., Heinsch, M., Betts, D., Booth, D., & Kay-Lambkin, F. (2021). Barriers and facilitators to the use of e-health by older adults: a scoping review. *BMC public health*, *21*, 1-12.

41. Alruwaili, M. M., Shaban, M., & Elsayed Ramadan, O. M. (2023). Digital health interventions for promoting healthy aging: a systematic review of adoption patterns, efficacy, and user experience. *Sustainability*, *15*(23), 16503.

42. Maskell, K., McDonald, P., & Paudyal, P. (2018). Effectiveness of health education materials in general practice waiting rooms: a cross-sectional study. *British Journal of General Practice*, *68*(677), e869-e876.

43. Frohlich, D. M. (2017, October). From Audio Paper to Next Generation Paper. In *Proceedings of the 23rd Brazillian Symposium on Multimedia and the Web* (pp. 9-10).

44. Frohlich D, Corrigan-Kavanagh E, Bober M, et al. The Cornwall a-book: An augmented travel guide using next generation paper. The Journal of Electronic Publishing 2019;22(1). DOI: https://doi.org/10.3998/3336451.0022.101.

45. Bairaktaris, G., Frohlich, D., & Sporea, R. (2021, May). Printed Light Tags and the Magic Bookmark: Using light to augment paper objects. In *Extended Abstracts of the 2021 CHI Conference on Human Factors in Computing Systems* (pp. 1-5).

46. Frohlich, D. M., Ferraz, L., & Castro, P. (2022). Printed digital storytelling for older audiences [2]

For peer review only

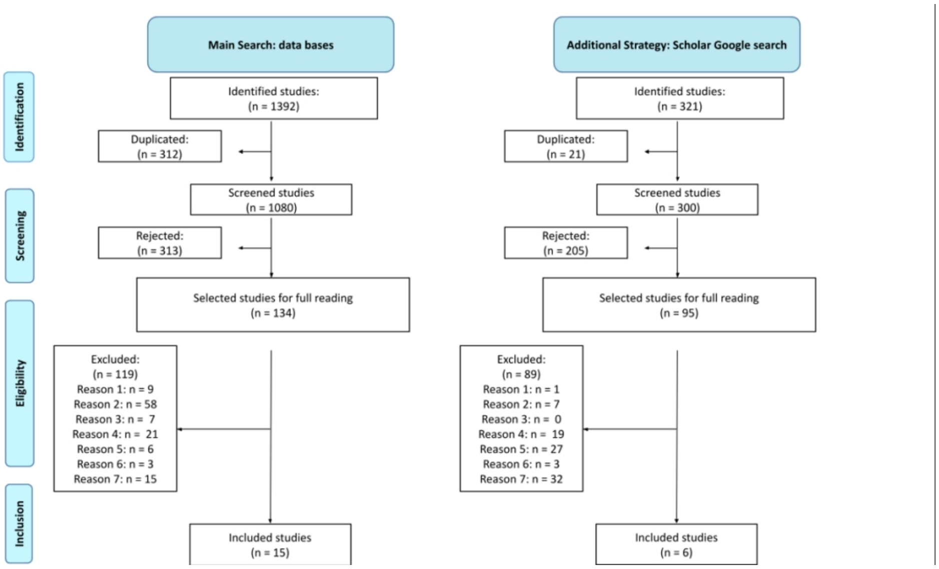

Description: Reason 1: it is a protocol, literature review or it has only an abstract available; Reason 2: it does not present the mean age or the mean age is less than 60 years; Reason 3: it does not have participants; Reason 4: it does not have a health education or the health education is not for promotion or prevention; Reason 5: health education does not use paper or screen-based media; Reason 6: it does not describe the health education; Reason 7: it does not present barriers or facilitators perceived by users in relation to the media used.
Source: developed by the authors.

234x131mm (144 x 144 DPI)

Database	Search Strategy
Scopus	TITLE-ABS-KEY (("older person" OR elder* OR "older adults" OR "elderly population" OR "older people" OR ageing OR aging OR "older population" OR geriatric OR "healthy ageing" OR "successful aging") AND ("health education" OR "health information" OR "health communication" OR "health promotion") AND ("paper based media" OR "screen based media" OR website* OR platform* OR virtual OR online OR multimodal OR multimedia OR "reading media" OR "digital-based reading" OR "paper based reading" OR flyer OR "media advertisement" OR "print media" OR app OR apps OR tablet* OR smartphone* OR m-health* OR e-health* OR "patient information leaflets") AND (preference* OR characteristic* OR experience* OR attribute* OR perception* OR development OR barrier* OR facilitator* OR opportunities OR problem* OR recommendations) AND (trial OR intervention))
Web of Science	TS=((“older person“ OR elder* OR “older adults” OR “elderly population” OR “older people” OR ageing OR aging OR “older population” OR geriatric OR “healthy ageing” OR “successful aging”) AND (“health education“ OR “health information” OR “health communication” OR “health promotion”) AND (“paper based media” OR “screen based media” OR website* OR platform* OR virtual OR online OR multimodal OR multimedia OR “reading media” OR “digital-based reading” OR “paper based reading” OR flyer OR “media advertisement” OR “print media” OR app OR apps OR tablet* OR smartphone* OR m-health* OR e-health* OR “patient information leaflets”) AND (preference* OR characteristic* OR experience* OR attribute* OR perception* OR development OR barrier* OR facilitator* OR opportunities OR problem* OR recommendations) AND (trial OR intervention))
Medline	AB ((“older person“ OR elder* OR “older adults” OR “elderly population” OR “older people” OR ageing OR aging OR “older population” OR geriatric OR “healthy ageing” OR “successful aging”) AND (“health education“ OR “health information” OR “health communication” OR “health promotion”) AND (“paper based media” OR “screen based media” OR website* OR platform* OR virtual OR online OR multimodal OR multimedia OR “reading media” OR “digital-based reading” OR “paper based reading” OR flyer OR “media advertisement” OR “print media” OR app OR apps OR tablet* OR smartphone* OR m-health* OR e-health* OR “patient information leaflets”) AND (preference* OR characteristic* OR experience* OR attribute* OR perception* OR development OR barrier* OR facilitator* OR opportunities OR problem* OR recommendations) AND (trial OR intervention))
Embase	('older person':ti,ab,kw OR elder*:ti,ab,kw OR 'older adults':ti,ab,kw OR 'elderly population':ti,ab,kw OR 'older people':ti,ab,kw OR ageing:ti,ab,kw OR aging:ti,ab,kw OR 'older population':ti,ab,kw OR geriatric:ti,ab,kw OR 'healthy ageing':ti,ab,kw OR 'successful aging':ti,ab,kw) AND ('health education':ti,ab,kw OR 'health information':ti,ab,kw OR 'health communication':ti,ab,kw OR 'health promotion':ti,ab,kw) AND ('paper based media':ti,ab,kw OR 'screen based media':ti,ab,kw OR website*:ti,ab,kw OR platform*:ti,ab,kw OR virtual:ti,ab,kw OR online:ti,ab,kw OR multimodal:ti,ab,kw OR multimedia:ti,ab,kw OR 'reading media':ti,ab,kw OR 'digital-based reading':ti,ab,kw OR 'paper based reading':ti,ab,kw OR flyer:ti,ab,kw OR 'media advertisement':ti,ab,kw OR 'print media':ti,ab,kw OR app:ti,ab,kw OR apps:ti,ab,kw OR tablet*:ti,ab,kw OR smartphone*:ti,ab,kw OR 'm health*':ti,ab,kw OR 'e health*':ti,ab,kw OR 'patient information leaflets':ti,ab,kw) AND (preference*:ti,ab,kw OR characteristic*:ti,ab,kw OR experience*:ti,ab,kw OR attribute*:ti,ab,kw OR perception*:ti,ab,kw OR development:ti,ab,kw OR barrier*:ti,ab,kw OR facilitator*:ti,ab,kw OR opportunities:ti,ab,kw OR problem*:ti,ab,kw OR recommendations:ti,ab,kw) AND (trial:ti,ab,kw OR intervention:ti,ab,kw)

Cinahl	AB ((“older person“ OR elder* OR “older adults” OR “elderly population” OR “older people” OR ageing OR aging OR “older population” OR geriatric OR “healthy ageing” OR “successful aging”) AND (“health education“ OR “health information” OR “health communication” OR “health promotion”) AND (“paper based media” OR “screen based media” OR website* OR platform* OR virtual OR online OR multimodal OR multimedia OR “reading media” OR “digital-based reading” OR “paper based reading” OR flyer OR “media advertisement” OR “print media” OR app OR apps OR tablet* OR smartphone* OR m-health* OR e-health* OR “patient information leaflets”) AND (preference* OR characteristic* OR experience* OR attribute* OR perception* OR development OR barrier* OR facilitator* OR opportunities OR problem* OR recommendations) AND (trial OR intervention))
The ACM Guide to Computing Literature	[[Abstract: "older person"] OR [Abstract: elder*] OR [Abstract: "older adults"] OR [Abstract: "elderly population"] OR [Abstract: "older people"] OR [Abstract: ageing] OR [Abstract: aging] OR [Abstract: "older population"] OR [Abstract: geriatric] OR [Abstract: "healthy ageing"] OR [Abstract: "successful aging"]] AND [[Abstract: "health education"] OR [Abstract: "health information"] OR [Abstract: "health communication"] OR [Abstract: "health promotion"]] AND [[Abstract: "paper based media"] OR [Abstract: "screen based media"] OR [Abstract: website*] OR [Abstract: platform*] OR [Abstract: virtual] OR [Abstract: online] OR [Abstract: multimodal] OR [Abstract: multimedia] OR [Abstract: "reading media"] OR [Abstract: "digital-based reading"] OR [Abstract: "paper based reading"] OR [Abstract: flyer] OR [Abstract: "media advertisement"] OR [Abstract: "print media"] OR [Abstract: app] OR [Abstract: apps] OR [Abstract: tablet*] OR [Abstract: smartphone*] OR [Abstract: m-health*] OR [Abstract: e-health*] OR [Abstract: "patient information leaflets"]] AND [[Abstract: preference*] OR [Abstract: characteristic*] OR [Abstract: experience*] OR [Abstract: attribute*] OR [Abstract: perception*] OR [Abstract: development] OR [Abstract: barrier*] OR [Abstract: facilitator*] OR [Abstract: opportunities] OR [Abstract: problem*] OR [Abstract: recommendations]] AND [[Abstract: trial] OR [Abstract: intervention]]
PsycInfo (APA)	(“older person“ OR elder* OR “older adults” OR “elderly population” OR “older people” OR ageing OR aging OR “older population” OR geriatric OR “healthy ageing” OR “successful aging”) AND Abstract: (“health education“ OR “health information” OR “health communication” OR “health promotion”) AND Abstract: (“paper based media” OR “screen based media” OR website* OR platform* OR virtual OR online OR multimodal OR multimedia OR “reading media” OR “digital-based reading” OR “paper based reading” OR flyer OR “media advertisement” OR “print media” OR app OR apps OR tablet* OR smartphone* OR m-health* OR e-health* OR “patient information leaflets”) AND (Abstract: preference* OR Abstract: characteristic* OR Abstract: experience* OR Abstract: attribute* OR Abstract: perception* OR Abstract: development OR Abstract: barrier* OR Abstract: facilitator* OR Abstract: opportunities OR Abstract: problem* OR Abstract: recommendations) AND (Abstract: trial OR Abstract: intervention)

Preferred Reporting Items for Systematic reviews and Meta-Analyses extension for Scoping Reviews (PRISMA-ScR) Checklist

SECTION	ITEM	PRISMA-ScR CHECKLIST ITEM	REPORTED ON PAGE #
**TITLE**			
Title	1	Identify the report as a scoping review.	
**ABSTRACT**			
Structured summary	2	Provide a structured summary that includes (as applicable): background, objectives, eligibility criteria, sources of evidence, charting methods, results, and conclusions that relate to the review questions and objectives.	
**INTRODUCTION**			
Rationale	3	Describe the rationale for the review in the context of what is already known. Explain why the review questions/objectives lend themselves to a scoping review approach.	
Objectives	4	Provide an explicit statement of the questions and objectives being addressed with reference to their key elements (e.g., population or participants, concepts, and context) or other relevant key elements used to conceptualize the review questions and/or objectives.	
**METHODS**			
Protocol and registration	5	Indicate whether a review protocol exists; state if and where it can be accessed (e.g., a Web address); and if available, provide registration information, including the registration number.	
Eligibility criteria	6	Specify characteristics of the sources of evidence used as eligibility criteria (e.g., years considered, language, and publication status), and provide a rationale.	
Information sources*	7	Describe all information sources in the search (e.g., databases with dates of coverage and contact with authors to identify additional sources), as well as the date the most recent search was executed.	
Search	8	Present the full electronic search strategy for at least 1 database, including any limits used, such that it could be repeated.	
Selection of sources of evidence†	9	State the process for selecting sources of evidence (i.e., screening and eligibility) included in the scoping review.	
Data charting process‡	10	Describe the methods of charting data from the included sources of evidence (e.g., calibrated forms or forms that have been tested by the team before their use, and whether data charting was done independently or in duplicate) and any processes for obtaining and confirming data from investigators.	
Data items	11	List and define all variables for which data were sought and any assumptions and simplifications made.	
Critical appraisal of individual sources of evidence§	12	If done, provide a rationale for conducting a critical appraisal of included sources of evidence; describe the methods used and how this information was used in any data synthesis (if appropriate).	
Synthesis of results	13	Describe the methods of handling and summarizing the data that were charted.	

For peer review only

SECTION	ITEM	PRISMA-ScR CHECKLIST ITEM	REPORTED ON PAGE #
**RESULTS**			
Selection of sources of evidence	14	Give numbers of sources of evidence screened, assessed for eligibility, and included in the review, with reasons for exclusions at each stage, ideally using a flow diagram.	
Characteristics of sources of evidence	15	For each source of evidence, present characteristics for which data were charted and provide the citations.	
Critical appraisal within sources of evidence	16	If done, present data on critical appraisal of included sources of evidence (see item 12).	
Results of individual sources of evidence	17	For each included source of evidence, present the relevant data that were charted that relate to the review questions and objectives.	
Synthesis of results	18	Summarize and/or present the charting results as they relate to the review questions and objectives.	
**DISCUSSION**			
Summary of evidence	19	Summarize the main results (including an overview of concepts, themes, and types of evidence available), link to the review questions and objectives, and consider the relevance to key groups.	
Limitations	20	Discuss the limitations of the scoping review process.	
Conclusions	21	Provide a general interpretation of the results with respect to the review questions and objectives, as well as potential implications and/or next steps.	
**FUNDING**			
Funding	22	Describe sources of funding for the included sources of evidence, as well as sources of funding for the scoping review. Describe the role of the funders of the scoping review.	

JBI = Joanna Briggs Institute; PRISMA-ScR = Preferred Reporting Items for Systematic reviews and Meta-Analyses extension for Scoping Reviews.

* Where *sources of evidence* (see second footnote) are compiled from, such as bibliographic databases, social media platforms, and Web sites.

† A more inclusive/heterogeneous term used to account for the different types of evidence or data sources (e.g., quantitative and/or qualitative research, expert opinion, and policy documents) that may be eligible in a scoping review as opposed to only studies. This is not to be confused with *information sources* (see first footnote).

‡ The frameworks by Arksey and O'Malley (6) and Levac and colleagues (7) and the JBI guidance (4, 5) refer to the process of data extraction in a scoping review as data charting.

§ The process of systematically examining research evidence to assess its validity, results, and relevance before using it to inform a decision. This term is used for items 12 and 19 instead of "risk of bias" (which is more applicable to systematic reviews of interventions) to include and acknowledge the various sources of evidence that may be used in a scoping review (e.g., quantitative and/or qualitative research, expert opinion, and policy document).

From: Tricco AC, Lillie E, Zarin W, O'Brien KK, Colquhoun H, Levac D, et al. PRISMA Extension for Scoping Reviews (PRISMAScR): Checklist and Explanation. Ann Intern Med. 2018;169:467–473. doi: 10.7326/M18-0850.

For peer review only - http://bmjopen.bmj.com/site/about/guidelines.xhtml
